# Contrasting gene expression patterns during floral induction in two *Chenopodium ficifolium* genotypes reveal putative flowering regulators

**DOI:** 10.1080/15592324.2025.2486083

**Published:** 2025-04-04

**Authors:** David Gutiérrez-Larruscain, Manuela Krüger, Oushadee A. J. Abeyawardana, Claudia Belz, Petre I. Dobrev, Radomíra Vaňková, Kateřina Eliášová, Zuzana Vondráková, Miloslav Juříček, Helena Štorchová

**Affiliations:** Institute of Experimental Botany, Czech Academy of Sciences, Prague, Czech Republic

**Keywords:** Flowering, long-day *Chenopodium ficifolium*, transcriptome, phytohormones, genes with contrasting expression trends

## Abstract

*Chenopodium ficifolium* is a close diploid relative of the tetraploid crop *Chenopodium quinoa*. Owing to its reproducible germination and seedling development, it becomes a promising model for studying floral induction, providing a basis for the comparison with *C. quinoa*. Two *C. ficifolium* genotypes differ in photoperiodic requirement: *C. ficifolium* 283 accelerates flowering under long days, whereas *C. ficifolium* 459 flowers earlier under short days. This study conducted a comprehensive transcriptomic and hormonomic analysis of floral induction in the long-day *C. ficifolium* 283 and compared the findings to previous experiments with the short-day *C. ficifolium*. Phytohormone concentrations and gene expression profiles during floral induction were largely similar between the two genotypes. However, a subset of genes exhibited contrasting expression patterns, aligning with the genotypes’ differing photoperiodic requirements. These genes, predominantly homologs of flowering-related genes in *Arabidopsis thaliana*, were activated under long days in *C. ficifolium* 283 and under short days in *C. ficifolium* 459. Notably, the contrasting expression of the *FLOWERING LOCUS T-LIKE 2–1* gene, which was previously shown to induce precocious flowering in *A. thaliana*, confirmed its role as a floral activator, despite its low expression levels.

## 1. Introduction

The transition from the vegetative to the reproductive phase represents a pivotal commitment in the life cycle of all plant species, holding particular significance for weeds. Weeds must grow and reproduce quickly in freshly disturbed areas, where competition with other plants is temporarily limited.^1^ It is advantageous for them to flower as early as possible because it ensures the production of at least some seeds by the end of summer or within small openings among robust grass tussocks. In contrast, postponing flowering until early summer while accumulating resources allows for the production of a greater number of seeds.

The induction of flowering is regulated by complex genetic signaling pathways that are integrated by essential floral regulators, such as the *FLOWERING LOCUS T* (*FT*) genes.^2,3^ Through repeated duplications in the course of evolution, these genes have undergone subfunctionalizations or neofunctionalizations, enabling fine-tuning of regulatory circuits to ensure the optimal timing of flowering. An excellent example is the pair of floral activators, *FT* and *TWIN SISTER OF FT* (*TSF*), in *Arabidopsis thaliana*. *FT* promotes flowering under long-day conditions (hereafter LD),^4^ while *TSF* functions under non-inductive short-day conditions (hereafter SD).^5^ *FT* paralogs may even acquire the opposite function of floral inhibition, as seen in sugar beet, where the *BvFT2* gene activates flowering while *BvFT1* suppresses it.^6^ While genetic pathways regulating flowering have been extensively studied in several model plants and crops,^7,8^ pertinent information remains scarce for a vast majority of wild species, including weeds.

The *Chenopodiaceae* family includes both crops and numerous weedy species. Floral induction has been investigated in sugar beet,^6^ *Chenopodium quinoa* Willd,^9^ and the weeds *Oxybasis rubra* (L.) S. Fuentes, Uotila & Borsch [≡*C. rubrum* L.]^10^ and *Chenopodium ficifolium* Sm.^11–14^ FT homologs were activated during flowering in all these plant models, with one exception - *C. ficifolium* 283. This particular accession, collected in Central Europe, exhibited accelerated flowering under LD, despite the notably low expression of all three *FT* paralogs.^14^ In contrast, another accession, *C. ficifolium* 459, collected in Central Asia, flowered earlier under SD, concomitant with the activation of *CfFTL1*.^12,14^ The existence of two conspecific genotypes with distinct photoperiodic responses and apparently different flowering regulation system makes *C. ficifolium* an ideal model for a comprehensive comparative study of floral induction. As a close diploid relative of tetraploid *C. quinoa*, *C*. *ficifolium* contributes to our understanding of flowering regulation in this crop.

The comprehensive transcriptomic and hormonic study of *C. ficifolium* 459 confirmed the activation of the floral inducer *CfFTL1* at SD and found high concentrations of stress-related phytohormones under LD, when flowering was delayed.^12^ The similar comprehensive analysis of the genotype *C. ficifolium* 283 with accelerated flowering at LD was still missing. Here, we investigated transcriptomic and hormonal dynamics in the course of floral induction in this genotype. The comparison between *C. ficifolium* 283 and *C. ficifolium* 459 revealed the genes activated during floral induction and exhibiting the opposite trends at SD and LD. The most noteworthy was that the *CfFTL2–1* gene was only slightly expressed, but its transcription clearly correlated with flowering in a respective genotype. This gene acted as a very strong activator of flowering when expressed in *A. thaliana*.^11^ Therefore, it should be considered the floral activator in *C. ficifolium* 283.

## 2. Material and methods

### 2.1. Plant material and experimental conditions

The accession *C. ficifolium* 283 collected in the Czech Republic^14^ was used to follow floral induction. The same cultivation conditions as for *C. ficifolium* 459^12^ were applied to make the comparison possible. The plants were cultivated in the IEB greenhouse and propagated by self-pollination. Floral induction experiments were performed with seedlings grown in 96-well flat-bottom ELISA plates, single plantlet per well; soaked in half-strength Hoagland solution; and placed in Percival E-36L2 (22°C, 70% humidity, cool-white fluorescent light 130 μmol m^−2^ s^−1^, or dark). Two photoperiodic regimes were utilized: SD (6 h light and 18 h dark) and LD (18 h light and 6 h dark), following exactly the same experimental design as it was used with *C. ficifolium* 459.^12^

To estimate morphological parameters, the images of the whole seedlings, isolated cotyledons, and leaves were examined under Navitar Machine Vision (Navitar Inc., Rochester, NY, USA). The length of the shoot apex and flowering rate were assessed under the stereomicroscope Zeiss Stemi 305. The rate of flowering (in %) was determined as the proportion of plants with terminal flower bud in the whole set of tested plants). The images were recorded using a DFK 33UX250 camera (the Imaging Source, Bremen, Germany) and processed using NIS-Elements 5.0 (Laboratory Imaging, Prague, Czech Republic). About six plants from the specific photoperiodic regime (LD or SD) were measured.

### 2.2. RNA extraction and RT qPCR

The seedlings were sampled twice a day (in the morning at 9.00 and in the afternoon at 15.00) and in the days 13, 15, and 19 after sowing (DAS) under both SD and LD. Above-ground parts of the plantlets from each photoperiodic regime were taken and flash-frozen in liquid nitrogen. Three biological replicates consisted of three to five seedlings from LD conditions and eight to ten seedlings from SD conditions and were collected at each time point. Total RNA was isolated using a Plant RNeasy Mini kit (Qiagen, Valencia, CA, USA) exactly as described by Gutiérrez-Larruscain et al.^12^ RNA concentration and quality were checked on 0.9% agarose gel and using the NanoDrop (Thermo Fisher Scientific, Vantaa, Finland).

To prepare cDNA, 1 µg of RNA and oligo dT primers (500 ng) were heated for 5 min at 65°C; put on ice; and mixed with Transcriptor buffer (Roche), 0.5 μl of Protector RNase Inhibitor (Roche, Diagnostics, Mannheim, Germany), 2 μl of 10 mM dNTPs, and 10 units of Transcriptor Reverse Transcriptase (Roche). The first strand of cDNA was produced at 55°C for 30 min. Quantitative PCR was performed using the LightCycler 480 SYBR Green I Master (Roche) in a final volume of 10 μl with 500 nM of each of the primers. The *ACTIN11* (*ACT11*) gene was employed as the reference. The primers for the reference and target genes were described by Štorchová et al.^14^

### 2.3. Phytohormone measurements

Aerial parts of the seedlings for phytohormone concentration measurements were sampled simultaneously with the samples for RNA extraction flash-frozen in liquid nitrogen. Three biological replicates (about 20 mg of fresh weight) were taken at each sampling point. The samples were stored at −80°C until the start of phytohormone analyses. The endogenous phytohormone concentrations were determined following the protocols described by Gutiérrez-Larruscain et al.^12^ from about 20 mg of fresh weight (FW) (5–7 seedlings) per sample. Briefly, samples were extracted with 50% acetonitrile/water (1/1, v/v) and a mixture of internal standards was added. After purification using the SPE Oasis HLB plate, phytohormones were quantified by the LC/MS system consisting of UHPLC coupled to a Triple Quadrupole mass spectrometer.

### 2.4. Transcriptome assembly and evaluation

Total RNA was extracted from the seedlings collected at six time points under SD and LD and from the leaves, flowers, and roots of adult plants (39 RNA samples in total). The RNA specimens were sent to Macrogen (Seoul, Korea), where strand-specific cDNA libraries were constructed after polyA enrichment. The sequencing on the Illumina NovaSeq6000 produced 712 million raw paired-end (PE) reads (150 nt) altogether, about 18.7 million reads per specimen. Error correction, ribosomal RNA filtering, and quality trimming (cutoff 145 nt) were performed as described previously,^12^ and left 547 million trimmed pair-end reads. The raw and resulting trimmed data were stored under the BioProject number PRJNA891916 with SRA accessions SRR21998980–SRR21999010 for the raw reads and accessions SRR22032070–SRR22032100 for the trimmed reads.

Eleven transcriptomic sets (about 550 milions of trimmed reads) were *de novo*–assembled with Trinity v.2.9.0.^15^ The redundancy of the assembly was decreased by CD-Hit (similarity cutoff 99.9%) and then with the script EvidentialGene tr2aacds.pl (MINCDS = 50). The BUSCO^16^ search found 92% complete groups and the decrease of duplicated groups from 45% in the original Trinity assembly to 4% after the EvidentialGene application. The number of Trinity ‘genes’ dropped down to 35,565 in the EvidentialGene assembly. This set was used for a Blastx (BLAST +2.9.0) search against the nr database The cutoff E-value was set to *<*10^−4,^ and the maximum number of allowed hits was 10. The results were imported into the MEGAN pipeline^17^ and only plant hits were maintained. This Transcriptome Shotgun Assembly project has been deposited in DDBJ/EMBL/GenBank under the accession GKDG00000000. The version described in this paper is the first version, GKDG01000000.

### 2.5. Transcriptome annotation, read mapping and DEG identification

The *de novo* assembled transcriptome constructed from *C.* *ficifolium* 283 (GKDG01000000) contained 21,670 predicted Trinity ’genes’ and 25,671 predicted Trinity ‘transcripts’. The OmicsBox program v. 1.3.3 (BioBam Bioinformatics S.L., Valencia, Spain) was then used to annotate the Trinity ‘genes’ based on gene ontology (GO) terms, InterProScan, and nr database annotation. As *C. ficifolium* is closely related to *C. quinoa*, all the predicted Trinity ‘genes’ often gave blastx hits with the *C. quinoa* annotated genome.^18^ 75% of the ‘genes’ were GO (Gene Ontology) annotated, while 5% of the ‘genes’ were GO mapped.

The clean Illumina reads produced in the current floral induction experiment with *C. ficifolium* 283 were mapped against the *de novo* transcriptome. The transcript coverage was analyzed with the Trinity pipeline using the alignment-free method Salmon,^19^ as described in Krüger et al.^20^ The differential gene expression analysis was performed with the Bioconductor package DESeq2,^21^ three biological replicates for each sampling time point. Detection of differentially expressed genes (DEGs) was done for each sampling point, comparing the LD with the SD conditions with 0.05 cutoff for the false-discovery rate (FDR). The resulting DEGs and the log2fold change (logFC) values were used further.

The same reference transcriptome was also used to map the trimmed Illumina reads obtained during the previous floral induction experiment with *C. ficifolium* 459.^12^ About 85% of the reads from *C. ficifolium* 283 and a similar proportion of the reads of *C. ficifolium* 459 were mapped against this reference.

### 2.6. *The search for the genes with the contrasting expression pattern between* C. ficifolium *283 and* C. ficifolium *459*

In *C. ficifolium* 283, the genes involved in floral induction should be upregulated under LD conditions. In contrast, in *C. ficifolium* 459, we expect these genes to be upregulated under SD conditions, according to each photoperiod requirements. Therefore, we were interested in the genes with contrasting responses to different photoperiods.

To reveal these expression tendencies, the trimmed Illumina reads generated in the floral induction experiment with *C. ficifolium* 459^12^ were mapped against the new *C. ficifolium* 283 reference transcriptome to compare the gene expression patterns during floral induction between the two accessions.

To account for diurnal gene expression variations, we separately analyzed morning and afternoon expression data, resulting in “morning” and “afternoon” datasets.

We compared the second and third sampling points in *C. ficifolium* 283 (day 15 and day 19) and the third and fourth sampling points in *C. ficifolium* 459 (day 21 and day 24). These time points corresponded to the time window when the plantlets were either induced to flower under permissive conditions or not induced under non-permissive conditions.

To identify the genes with contrasting response to the photoperiod between the two *C. ficifolium* accessions, we extracted logFC values for differentially expressed genes (DEGs) from the pairwise comparisons between LD and SD regimes at the specific time points. We ranked these DEGs based on their logFC values, from highest (more highly expressed in LD) to lowest (more highly expressed in SD) in *C. ficifolium* 283 and *vice versa* in *C. ficifolium* 459. We assigned rank numbers to these genes, starting with 1 for the most highly differentially expressed gene.

Finally, we summed the rank numbers from the two sampling points (second and third in *C. ficifolium* 283, and third and fourth in *C. ficifolium* 459). Genes were then reordered based on this sum, from lowest to highest. Genes with the lowest rank numbers exhibited the most contrasting response to photoperiod between the two accessions. They were activated by LD in *C. ficifolium* 283 and, conversely, by SD in *C. ficifolium* 459, in line with each accession’s photoperiod requirement for flowering.

## 3. Results

### 3.1. *The comparison of the growth and floral transition between* C. ficifolium *283 and* C. ficifolium *459*

**Figure 1.**
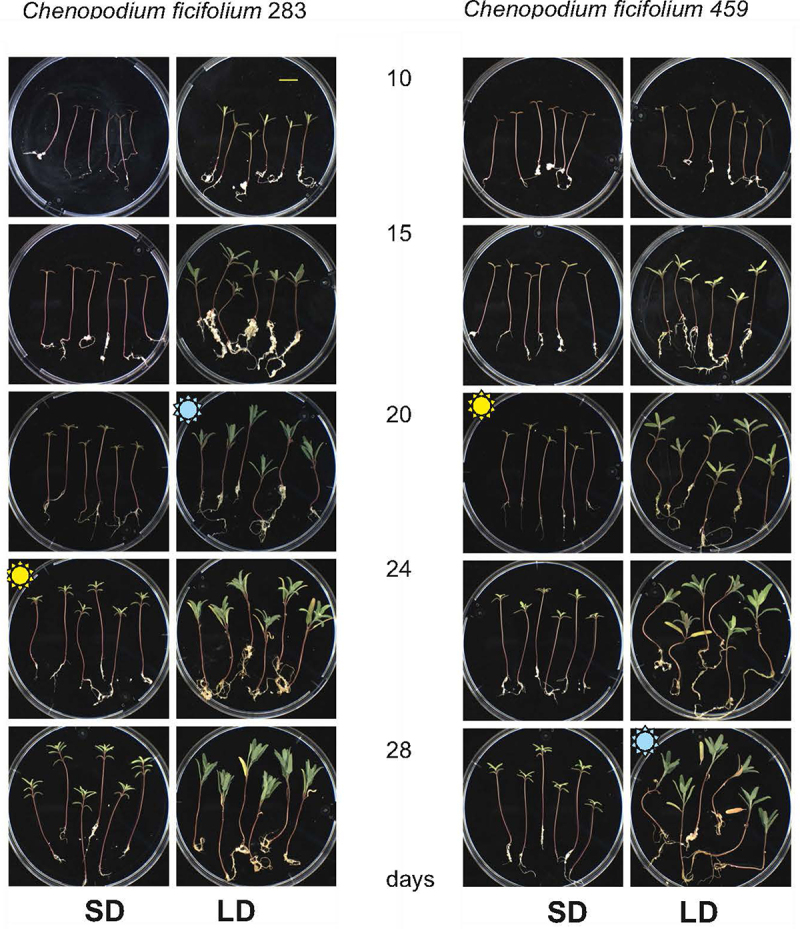
The comparison of *C. ficifolium* 283 and *C. ficifolium* 459. Seedlings were cultivated under short (SD) and long days (LD) at the ages 10 to 28 days after sowing (DAS). The yellow flower sign marks the onset of flowering at SD, and the blue flower signs marks the onset of flowering at LD.

**Figure 2.**
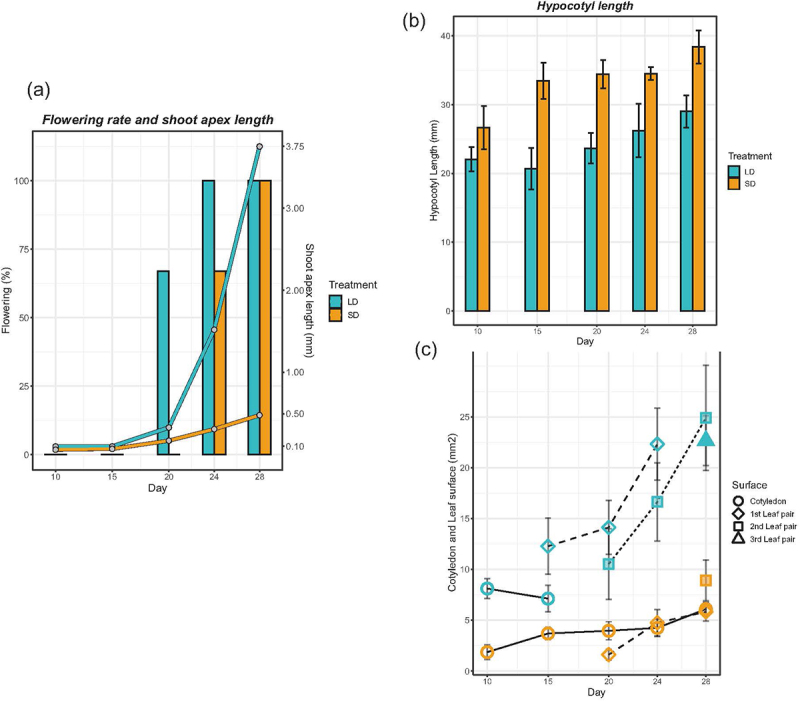
(a) The shoot apex length and proportion of the plants of *C. ficifolium* 283 with visible flower buds at the age 10 to 28 DAS at LD and SD. (b) The hypocotyl length in *C. ficifolium* at the ages of 10 to 28 DAS at LD and SD. (c) The cotyledon and leaf surface of *C. ficifolium* at the ages of 10 to 28 DAS. The third leaf pair (triangle) appeared under LD only. The cotyledons were lost before the age 20 DAS under LD, but persisted under SD.

The morphological and anatomical changes in seedlings were closely monitored during floral induction for both *C. ficifolium* 283 and *C. ficifolium* 459. These two accessions were cultivated and induced to flowering under identical conditions.^12^ Notably, the growth patterns of both *C. ficifolium* genotypes were highly similar (Figure 1). Under SD conditions, the plantlets of *C. ficifolium* 283 exhibited a slender and elongated hypocotyl (Figure 2b). However, they became more robust and developed larger leaves under LD (Figure 2c), similar to the growth of *C. ficifolium* 459 conditions.^12^

Nevertheless, the two genotypes diverged in terms of the onset of flowering, as noted in previously.^14^ Specifically, *C. ficifolium* 283 initiated flowering at about 20 days after sowing (DAS) under LD conditions but delayed by approximately four days under SD (Figure 1). This consistent delay in flowering under SD, as compared to LD, was confirmed through three independent experiments (Figure 2a). In contrast, the flowering of C. ficifolium 459 was accelerated at SD.^12^

### 3.2. *Phytohormone contents and phytohormone related gene expression in* C. ficifolium *283 compared to* C. ficifolium *459*

**Figure 3.**
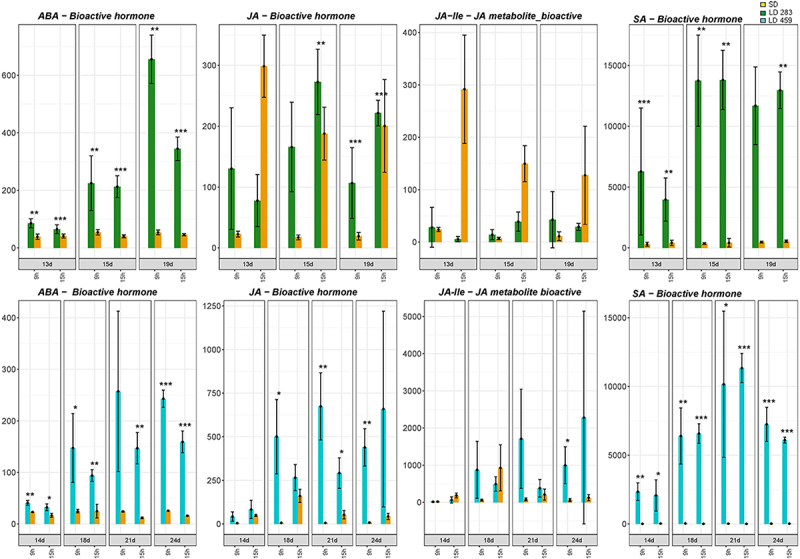
The concentrations of abscisic acid (ABA), jasmonic acid (JA), ja-isoleucine (JA-Ile), and salicylic acid (SA) in *C.* *ficifolium* 283 at the ages of 13 to 19 DAS and in *C. ficifolium* 459 at the ages of 14 to 24 DAS. Green or blue columns correspond to long LD treated samples, and golden ones represent SD treated samples. Transverse lines at each dot (median value of three biological replicates) represent standard deviation. Statistical significance (*p*-values * < 0.05, ** < 0.01, and ***< 0.001; t-test; three biological replicates, each consisting of 5 to 7 seedlings) between pairs of differentially treated samples is represented by asterisks. The x-axis represents sampling points (two sampling points per day: morning − 9.00 and afternoon − 15.00). The y-axis represents phytohomone concentration measured as pmol per gram of fresh weight.

The content of phytohormones and the expression of genes related to phytohormone metabolism and signaling were examined in both *C. ficifolium* 283 and *C. ficifolium* 459. Remarkably, the dynamics was either similar or identical between the two genotypes. There was a significant increase in the stress-related phytohormones abscisic acid (ABA), jasmonic acid (JA), and salicylic acid (SA) under LD conditions (Figure 3). Correspondingly, the expression levels of the genes responsible for the biosynthesis of ABA, JA, and SA also increased under LD in both genotypes (Supplementary Figures S1–S3).

**Figure 4.**
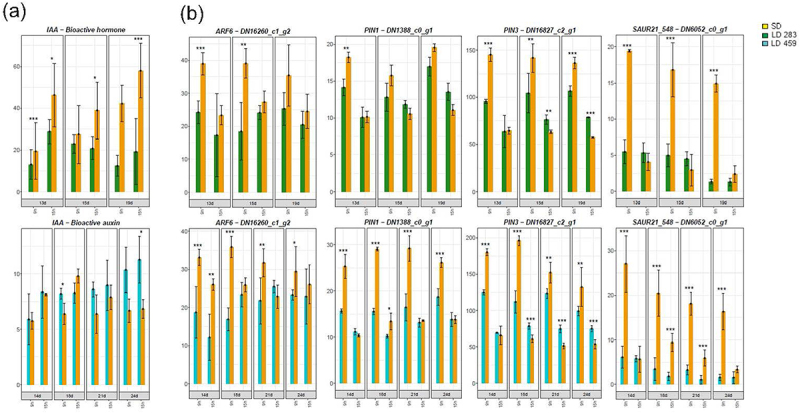
The auxin (AUX) concentrations and aux-related gene expression in *C. ficifolium 283* at the age 13 to 19 DAS and in *C. ficifolium* 459 at the age 14 to 24 DAS. Green or blue columns correspond to long LD-treated samples, golden ones represent SD-treated samples. Transverse lines at each dot (median value of three biological replicates) represent standard deviation. Statistical significance (*p*-values * < 0.05, ** < 0.01 and ***< 0.001; t-test; three biological replicates, each consisting of 5 to 7 seedlings) between pairs of differentially treated samples is represented by asterisks. The x-axis represents sampling points (two sampling points per day: morning − 9.00 and afternoon − 15.00). (a) Bioactive auxin (IAA) concentrations. The y-axis represents phytohomone concentration measured as pmol per gram of fresh weight. (b) The expression of *AUXIN RESPONSE FACTOR 6* (*ARF6*), *PIN-FORMED 1* (*PIN1*), *PIN-FORMED 3* (*PIN3*), and *SMALL AUXIN UPREGULATED RNA 21–548* (*SAUR21–548*). The y-axis represents relative expression in transcript coverage (TMM).

The bioactive auxin indol-3-acetic acid (IAA) concentration slightly increased at LD in *C*. *ficifolium* 283, while a similar slight elevation was observed at SD in *C. ficifolium* 459 (Figure 4a). Importantly, the expression profiles of auxin-related genes were highly similar in both genotypes (Figure 4b).

**Figure 5.**
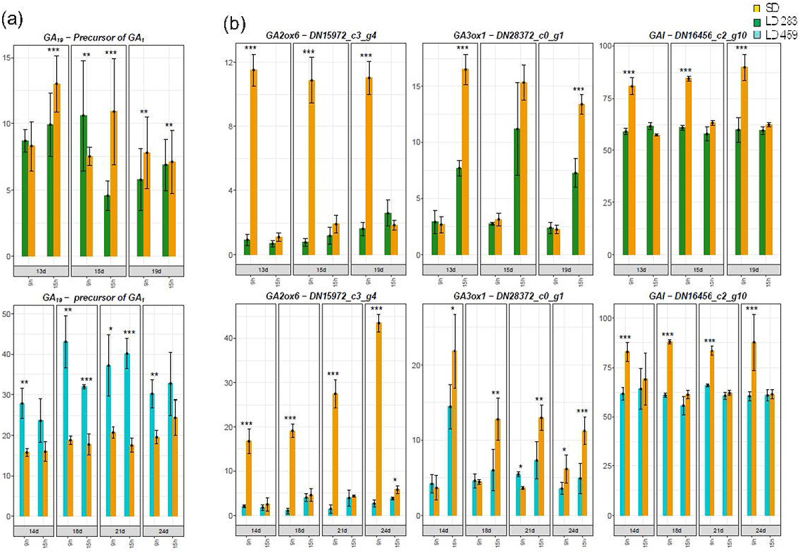
The gibberellic acid (GA) concentrations and GA-related gene expression in *C. ficifolium 283* at the age 13 to 19 DAS and in *C. ficifolium* 459 at the age 14 to 24 DAS. Green or blue columns correspond to long LD-treated samples, and golden ones represent SD-treated samples. Transverse lines at each dot (median value of three biological replicates) represent standard deviation. Statistical significance (*p*-values * < 0.05, ** < 0.01, and ***< 0.001; t-test; three biological replicates, each consisting of 5 to 7 seedlings) between pairs of differentially treated samples is represented by asterisks. The x-axis represents sampling points (two sampling points per day: morning − 9.00 and afternoon − 15.00). (a) GA_19_ concentrations. The y-axis represents phytohomone concentration measured as pmol per gram of fresh weight. (b) The expression of *GA2ox6* (involved in GA degradation), GA3ox1 (involved in GA biosynthesis), and *GIBBERELLIC ACID INSENSITIVE* (*GAI*). The y-axis represents relative expression in transcript coverage (TMM).

Because the concentrations of bioactive gibberellins in *C. ficifolium* 283 were below the detection limit, we only compared the levels of the precursor GA_19_ between the genotypes. We observed a slight elevation at LD in *C. ficifolium* 459, while no such increase was seen in *C. ficifolium* 283. The expression profiles of the gibberellin-related genes were closely aligned between the two genotypes (Figure 5).

Bioactive cytokinins (CKs) exhibited a slight increase under SD conditions in both genotypes (Figure 6a). The genes involved in CK metabolism also displayed increased expression (Supplementary Figure S4, S5). Interestingly, the concentration profiles of some CK derivatives differed between the genotypes. The concentration of the isopentenyl adenosine (iP) precursor isopentenyl adenosine monophosphate (iPRMP) was 10 times higher under LD in *C. ficifolium* 283 than in the 459 genotype, which correlated well with floral induction accompanied by promotion of cell division. Similarly, the levels of CK precursors were much higher under LD in *C. ficifolium* 283 (Figure 6b).

**Figure 6.**
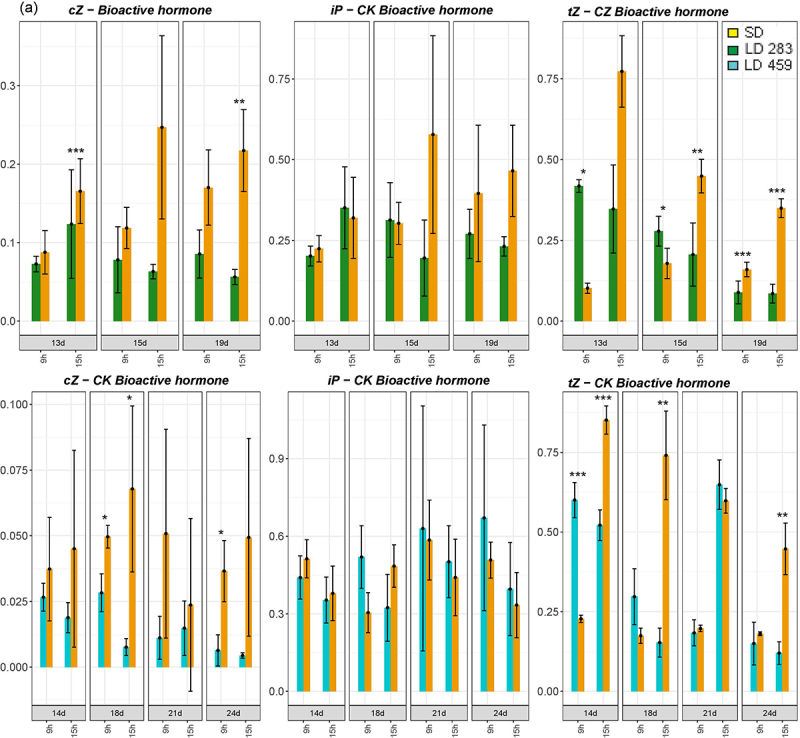
The cytokinin (CK) concentrations in *C. ficifolium 283* at the age 13 to 19 DAS and in *C. ficifolium* 459 at the age 14 to 24 DAS. Green or blue columns correspond to long LD-treated samples, and golden ones represent SD-treated samples. Transverse lines at each dot (median value of three biological replicates) represent standard deviation. Statistical significance (*p*-values * < 0.05, ** < 0.01, and ***< 0.001; t-test; three biological replicates, each consisting of 5 to 7 seedlings) between pairs of differentially treated samples is represented by asterisks. The x-axis represents sampling points (two sampling points per day: morning − 9.00 and afternoon − 15.00). The y-axis represents phytohomone concentration measured as pmol per gram of fresh weight. (a) The concentrations of bioactive CKs: *cis*-zeatin (*c*Z), isopentenyladenine (iP), and *trans*-zeatin (*t*Z). (b) The concentrations of CK derivatives: *trans*-zeatin *N7*-glucoside (*t*Z7G), *trans*-zeatin *O-*glucoside (*t*ZOG), *trans*-zeatin riboside (*t*ZR), isopentenyladenosine riboside (iPR), isopentenyl adenosine monophosphate (iPRMP), and 2-methylthiozeatin riboside (MeS-ZR).

**Figure 6.**
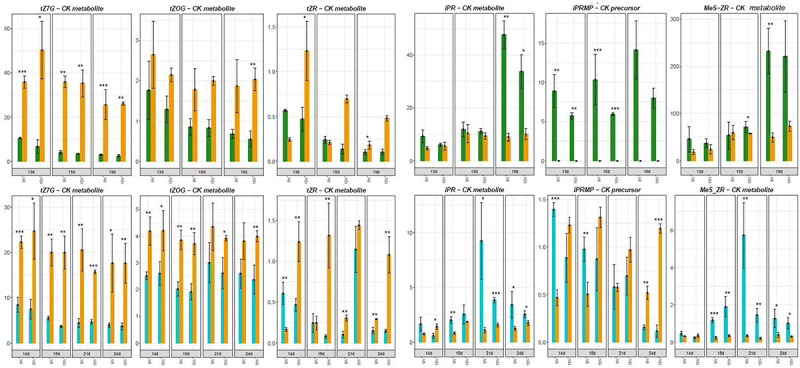
(Continued).

The overall concordance of gene expression profiles between the two *C. ficifolium* genotypes extended to other genes, for example, stress-related ones (Supplementary Figure S6). It greatly facilitated the identification of rare exceptions – the genes with opposite expression profiles, which could potentially play a role in the control of flowering.

### 3.3. *The identification of genes with contrasting expression pattern between* C. ficifolium *283 and* C. ficifolium *459*

The genes promoting floral induction in two genotypes with distinct photoperiodic requirements are expected to respond to the SD and LD in the opposite way. They should be activated by SD in *C. ficifolium* 459, but by LD in *C. ficifolium* 283. The profiles of the genes differentially expressed between the LD and SD in *C. ficifolium* 283 and *C. ficifolium* 459 were compared separately in the “morning” and “afternoon” datasets, and the genes with too low or fluctuating expression were excluded. 26 “morning” and 24 “afternoon” genes with contrasting expression profiles between the two genotypes were identified. As 15 genes exhibited opposite expression in both “morning” and “afternoon” datasets, we have finally identified 35 candidate genes (26 plus 24 minus 15), which might have been associated with floral induction (Table 1). Nine of them were the homologs of the flower development controlling genes – e.g. *LEAFY* (*LFY*) *APETALA 1* (*AP1)*, *AGAMOUS LIKE 9* (*AGL9*), or *AGAMOUS* (*AG*). Likewise, the *CfFTL2–1* gene was slightly upregulated at LD in *C. ficifolium 283* and at SD *C. ficifolium 459* (Figure 7). However, this activation was very weak compared with a hundredfold increase of *FTL1* at SD, which triggered flowering in *C. ficifolium 459*. The expression of the homolog of the floral integrator *SUPPRESSOR OF OVEREXPRESSION OF CO 1* (*SOC1*) was higher at LD in both genotypes, regardless of photoperiodic requirements. Not all homologs of flowering-related genes exhibited contrasting expression profiles. For example, *CAULIFLOWER* (*CAL*) was elevated at SD, and *APETALA 2* (*AP2*) was slightly activated at LD in both *C. ficifolium* genotypes (Figure 7).

**Figure 7.**
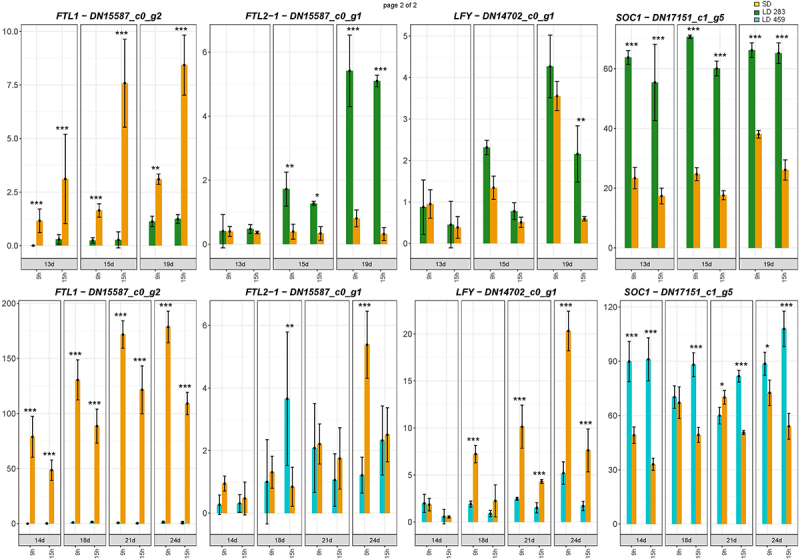
The flowering-related genes expressed in *C. ficifolium 283* at the age 13 to 19 DAS and in *C. ficifolium* 459 at the ages of 14 to 24 DAS. Green or blue columns correspond to long LD-treated samples, and golden ones represent SD-treated samples. Transverse lines at each dot (median value of three biological replicates) represent standard deviation. Statistical significance (*p*-values * < 0.05, ** < 0.01, and ***< 0.001; t-test; three biological replicates, each consisting of 5 to 7 seedlings) between pairs of differentially treated samples is represented by asterisks. The x-axis represents sampling points (two sampling points per day: morning − 9.00 and afternoon − 15.00). The y-axis represents relative expression in transcript coverage (TMM).

**Figure 7.**
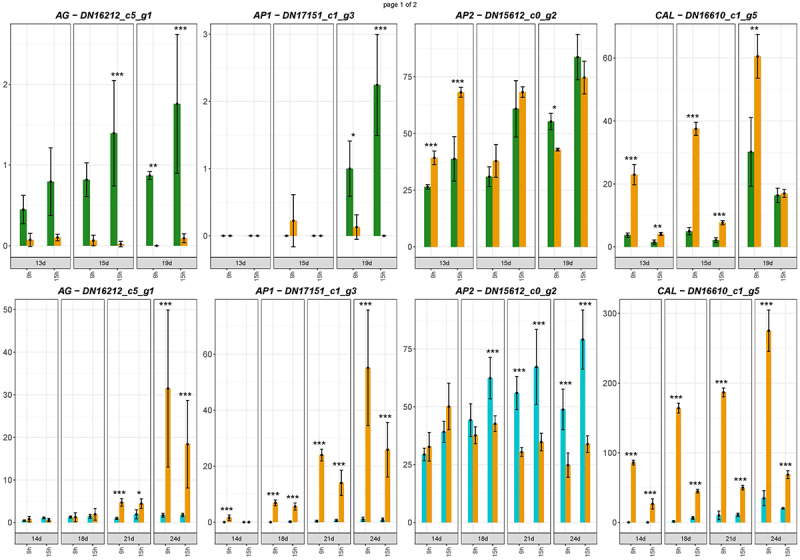
(Continued).

**Table 1.** The genes with contrasting expression pattern between *C. ficifolium* 283 and C. *ficifolium* 459.

Trinity gene ID	Blastx hit	Protein	Gene name	AGI code
		**floral induction and floral meristem specificity**		
DN15587_c0_g1	MK212027.1	FLOWERING LOCUS T-like 2–1	*FTL2–1*	AT1G65480
DN14702_c0_g1	XP_021715316.1	LEAFY	*LFY*	AT5G61850
DN16212_c5_g1	XP_021723464.1	AGAMOUS	*AG*	AT4G18960
DN15826_c1_g8	XP_021723077.1	AGAMOUS LIKE 9	*AGL9*	AT1G24260
DN15826_c1_g2	XP_021756511.1	MADS box CMB1 like	*CMB1*	AT5G15800
DN17151_c1_g3	XP_021756492.1	APETALA1	*AP1*	AT1G69120
DN17151_c1_g7	XP_021739401.1	APETALA1	*AP1*	AT1G69120
DN12941_c0_g1	XP_021759869.1	DEFICIENS-like	*AP3*	AT3G54340
DN6382_c0_g1	XP_021716384.1	CENTRORADIALIS-LIKE	*ATC*	AT2G27550
		**transcription factors**		
DN16786_c0_g1	XP_021726586.1	REM 16 LIKE	*REM16*	AT4G33280
DN5874_c0_g1	XP_021719303	SQUAMOSA PROMOTER BINDING-like 3	*SPL3*	AT2G33810
DN4836_c0_g1	XP_021721780.1	ALBA2	*ALBA2*	AT2G34160
DN6206_c0_g1	XP_021738539	PHD like	*PHD*	n.a.
		**receptors**		
DN16864_c2_g1	XP_021761979.1	STRUBBELIG-RECEPTOR FAMILY 5-like	*SRF5*	AT1G78980
DN17110_c1_g3	XP_021862911.1	G-type lectin S-receptor-like serine/threonine-protein kinase	*EGM1*	AT1G11300
DN17858_c1_g1	XP_021854253.1	TOO MANY MOUTHS-like	*TMM*	AT1G80080
		**transporters, enzymes, regulatory protein**s		
DN6472_c0_g1	XP_021766950.1	PPDK REGULATORY PROTEIN	*RP1*	AT4G21210
DN9403_c0_g1	XP_021774770.1	LYS/HIS Transporter	*n.a.*	AT1G47670
DN15894_c2_g2	XP_021730829.1	FORMIN LIKE 3	*FH5*	AT5G54650
DN16268_c2_g1	XP_021738705.1	SQUALENE SYNTHASE	*SQS1*	AT4G34640
DN17223_c2_g3	XP_021753867.1	SUBTILISIN-like protease	*SBT2.6*	AT2G19170
		**stress response**		
DN10374_c0_g1	XP_021725567.1	ALTERNATIVE OXIDASE 1	*AOX1*	AT3G22370
DN12254_c0_g1	XP_021769091.1	UDP-GLYCOSYLTRANFERASE 76B1 like	*UGT76B1*	AT3G11340
DN12047_c0_g1	XP_021723062.1	GAP JUNCTION BETA-4	*GJB4*	AT5G13100
DN16205_c2_g2	XP_021763941.1	HXXXD-type acyl-transferase	n.a.	AT3G50280
DN44685_c0_g1	XP_021744804.1	gamma-interferon-inducible lysosomal thiol reductase-like	*GILT*	AT4G12960
DN10404_c0_g1	XP_021749788.1	CYTOCHROME P450 71A	*CYP71A*	AT3G48310
DN16228_c2_g9	XP_021848616.1	putative 57 kDa heat shock protein	n.a.	AT4G16545
DN10919_c0_g1	XP_021748941.1	F-box kelch/repeat protein	n.a.	AT3G23880
DN13237_c0_g1	XP_021766921.1	F-box kelch/repeat protein	n.a.	AT3G23880
DN7988_c0_g1	XP_021758808.1	F-box kelch/repeat protein	n.a.	AT1G15680
DN14014_c0_g1	XP_021732566.1	ring canal kelch homolog	n.a.	no hit
		**hypothetical proteins**		
DN17090_c1_g1	KNA05366.1	hypothetical protein SOVF_191020	n.a.	no hit
DN34381_c0_g1	KMT14149.1	hypothetical protein BVRB_4g079700	n.a.	no hit
DN6395_c0_g1	XP_021714746.1	uncharacterized protein	n.a.	no hit
DN34018_c0_g1	XP_021736107.1	uncharacterized protein	n.a.	no hit

The rest of oppositely expressed genes coded for transcription factors, regulatory proteins, stress response-related proteins (e.g. coding for the homolog of *ALTERNATVE OXIDASE 1* (*AOX1*), or for uncharacterized proteins (Supplementary Figure S7).

### 3.4. The validation of gene expression by RT qPCR

The transcript levels of the *CfFTL1*, *CfFTL2–1*, and *CfFTL2–2* genes in the course of floral induction in *C. ficifolium* 283 were measured by RT qPCR (Supplementary Figure S8). The results agreed with the expression profiles estimated from the transcriptomic data. The *FTL2–2* gene is absent in *C. ficifolium* 459 and contains a large deletion in *C. ficifolium* 283. Its expression was analyzed only in the latter accession.

## 4. Discussion

### 4.1. *Reproducibility of floral induction experiments in the* C. ficifolium *genotypes*

The plantlets of *C. ficifolium* 283 grew in a similar way and pace as it was documented with *C. ficifolium* 459.^12^ The primary difference lay in the timing of flowering, which occurred more rapidly under LD in *C. ficifolium* 283, unlike opposite SD conditions in *C. ficifolium* 459. The general similarity between the genotypes existed also at the level of gene expression and phytohormone concentrations, where hundreds of identical or highly similar gene expression and phytohormone profiles were observed in both genotypes during floral induction.

The high reproducibility of the floral induction experiments conducted in young plantlets of *C. ficifolium* 283 and *C. ficifolium* 459 enabled the identification of the putative genes associated with the accleration of flowering in *C. ficifolium* 283 at LD. We expected that in such cases, gene expression or phytohormone concentrations would increase over time specifically in *C. ficifolium 283*, but not in *C. ficifolium* 459. To investigate this, we employed a newly developed algorithm, which identified 35 candidate genes with clearly opposite expression profiles between the two genotypes.

### 4.2. *The genes upregulated during floral induction in* C. ficifolium *283, but not in* C. ficifolium *459*

Among the essential integrators of flowering analyzed, only *CfFTL2–1* exhibited increased expression in line with floral induction under LD in *C. ficifolium 283*, while it increased under contrasting SD conditions in *C. ficifolium 459*. However, its maximum expression was approximately one hundredfold lower than the transcript level of *CfFTL1*, which activated flowering in *C.* *ficifolium*459.^12^ This leads us to question of whether such low transcription can induce flowering. Since *Chenopodium* is recalcitrant to transformation, direct evidence of the function of *CfFTL* genes is difficult to achieve. Instead, in the heterologous transformation of *A. thaliana*, the *CfFTL2–1* gene was adopted previously. *CfFTL2–1* overexpression was lethal and even slight activation of its expression controlled by an inducible promoter led to the early flowering in seedlings at cotyledon stage in *A. thaliana*.^11^ This suggests that the promoting effect of *CfFTL2–1* might be sufficient to induce flowering at low expression levels despite being not considered a floral activator previously due to its weak expression.^14^

The *CfFTL2–1* gene is the homolog of the sugar beet floral repressor *BvFT1*. The inhibitory function of BvFT1 is associated with unique amino acid substitutions: Asn (position 138), Gln (position 141), and Gln (position 142) in place of Tyr, Gly, and Trp in the respective positions of other FT floral activators.^6^ Unlike BvFT1, the CfFTL2–1 protein shares the aforementioned amino acids with other floral promoters. However, it possesses two unique substitutions that may be important for its function. It carries Pro in the position 145 and Ile in the position 148 instead of Gln and Asn in the corresponding positions in other floral inducers, including *A. thaliana* FT. Therefore, it is possible that these two amino acids highly activate the floral-inducing effect of CfFTL2–1.

The overexpression of the *CfFTL1* paralog in *A. thaliana* led to early flowering,^11^ but it could not induce flowering under LD as it was consistently upregulated under SD – strongly in *C. ficifolium* 459 and only slightly in *C. ficifolium* 283.

Another paralog, *CfFTL2–2*, which is present only in *C. ficifolium 283*, exhibited slight upregulation under LD conditions. However, its overexpression in *A. thaliana*^11^ had no effect, presumably due to a large deletion in the coding region.^14^ Consequently, it is unlikely that *CfFTL2–2* plays a role in floral promotion in *C.* *ficifolium* 283.

Several other transcription factors or regulatory genes were increasingly expressed during floral induction in both *C. ficifolium* genotypes, being potentially involved in the regulation of flowering and development. In addition to floral identity genes (i.e. *LFY*, *AP1*, *AG*), the *SQUAMOSA PROMOTER BINDING LIKE PROTEIN 3/4* (*SPL3/4*) homolog was noted. This gene has been reported to integrate photoperiodic and age-dependent signals in *A. thaliana*,^22^ and it may serve a similar role in *C. ficifolium*. In contrast, the expression of *SOC1*, the floral integrator in *A. thaliana*, was consistently higher at LD in both genotypes. Thus, it might have contributed to floral induction under LD in *C. ficifolium* 283, but not in *C. ficifolium* 459.

### 4.3. *Phytohormone concentrations in the two* C. ficifolium *genotypes responded to the photoperiods in a similar way*

LD conditions induced stress-related phytohormones ABA, JA, and SA in both *C. ficifolium* genotypes, regardless of whether flowering was accelerated or not. The occcurence of oxidative stress at LD was confirmed by the activation of many stress-response genes, such as those encoding peroxidases.^23^ There were some exceptions, e.g. the upregulation of the *ALTERNATIVE OXIDASE 1* (*AOX1*) homolog at LD in *C. ficifolium* 283, but at SD in *C. ficifolium* 283, which may be related to increased oxidative stress during floral development.^20^

The concentration profiles of bioactive CKs in both genotypes were slightly elevated at SD in both genotypes. Nevertheless, the high concentrations of some CK precursors and metabolites under LD conditions suggested a faster turnover in *C.* *ficifolium* 283. The increase of the biosynthesis of shoot-born CK iP, as indicated by high content of iPRMP, may be associated with the acceleration of flowering. Similarly, a slight increase in GA_19_ (a precursor of bioactive GA) or in auxin concentration at LD only in *C.* *ficifolium* 283 may suggest their involvement in floral development. As entire aerial parts of the plantlets were sampled for phytohormone concentration estimations, the obtained values represent the averages across plants. Detailed measurements of phytohormone concentration in individual organs or tissues should be performed in future to investigate the role of phytohormones in the flowering in *C. ficifolium*.

## 5. Conclusions

The expression profiles of the *CfFTL2–1* gene were found to correlate with floral induction in two *C. ficifolium* genotypes with contrasting response to photoperiod, but its transcript levels were low. However, this gene caused extraordinary strong induction of flowering, when expressed in *A. thaliana*.^11^ This suggests that *CfFTL2–1* may promote flowering in *C. ficifolium* 283 under LD when the floral activator *CfFTL1* is inhibited. The comprehensive transcriptomic and hormonomic study of floral induction was made possible by synchronous and reproducible seed germination and growth of this species. These features are exceptional in light of the reports on physiological and morphological variation in seed germination and growth in other *Chenopodium* species.^24^ *C. ficifolium* therefore emerges as a promising diploid model to study flowering in *Chenopodium* owing to its close relatedness to *C. quinoa* and also to its reproducible germination and growth.

## Supplementary Material

Suppl_Figures_Storchova_jpeg.zip

## Data Availability

The data may be accessed under the BioProject number PRJNA891916 with SRA accessions SRR21998980–SRR21999010 for the raw reads and accessions SRR22032070–SRR22032100 for the trimmed reads.
