# Supplementary figures and images for: Contrasting gene expression patterns during floral induction in two Chenopodium ficifolium genotypes reveal putative flowering regulators
Source: Plant Signal Behav. 2025 Apr 4;20(1):2486083. doi: 10.1080/15592324.2025.2486083 (PMC11980483; doi:10.1080/15592324.2025.2486083)

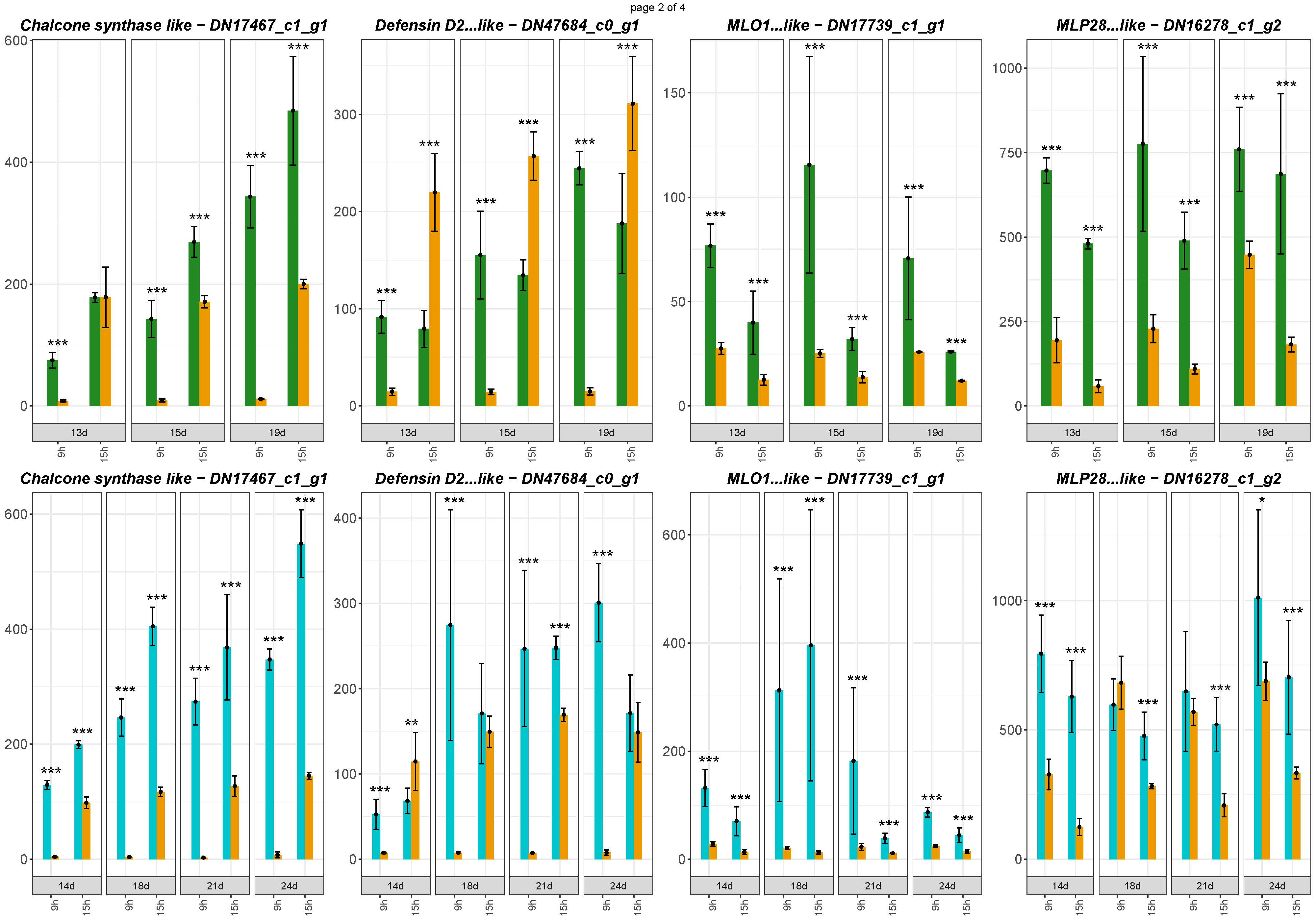

Supplement: Suppl_Figures_Storchova_jpeg.zip [file KPSB_A_2486083_SM1813.zip › Suppl_Figure6_Page_2.jpg]

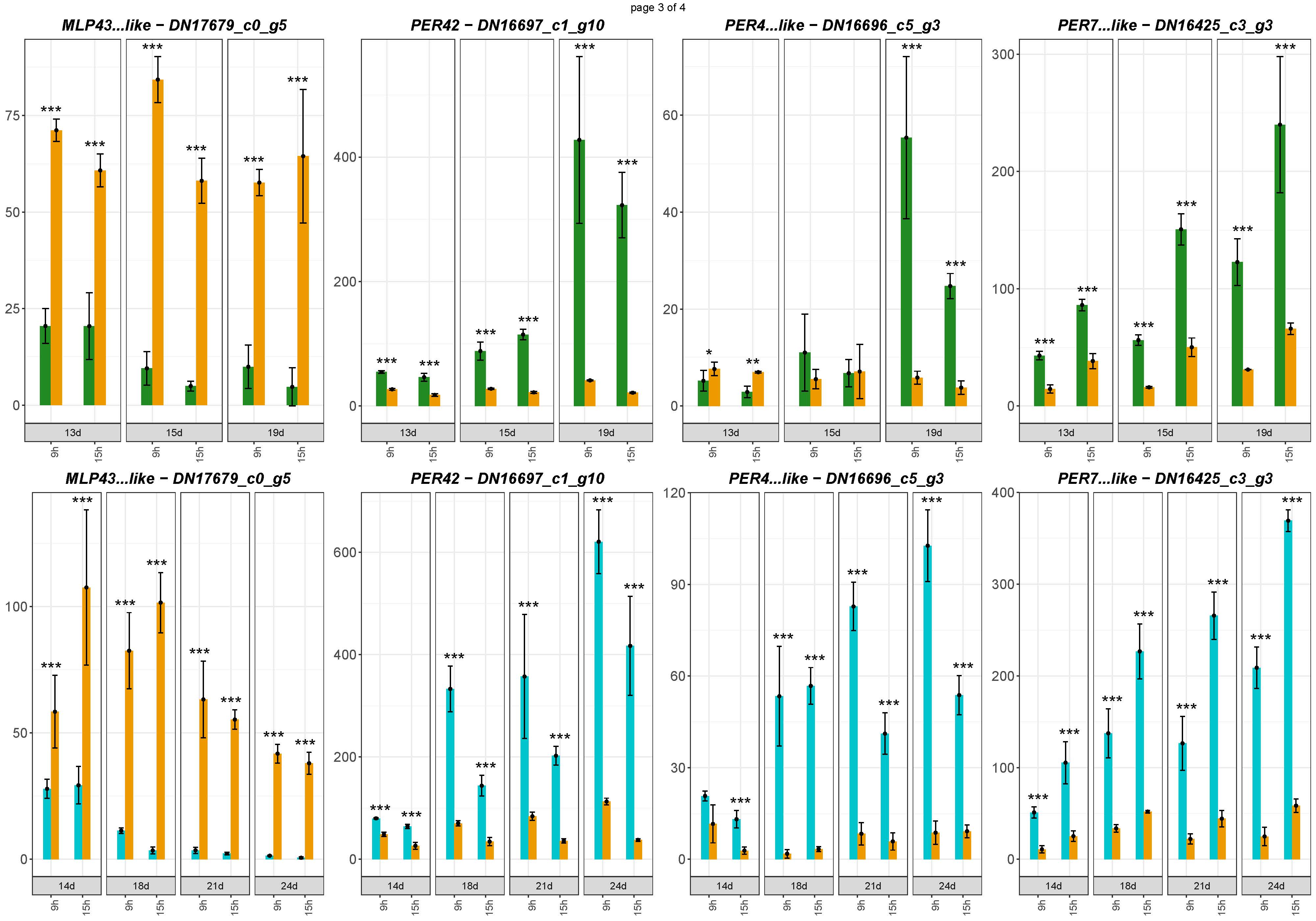

Supplement: Suppl_Figures_Storchova_jpeg.zip [file KPSB_A_2486083_SM1813.zip › Suppl_Figure6_Page_3.jpg]

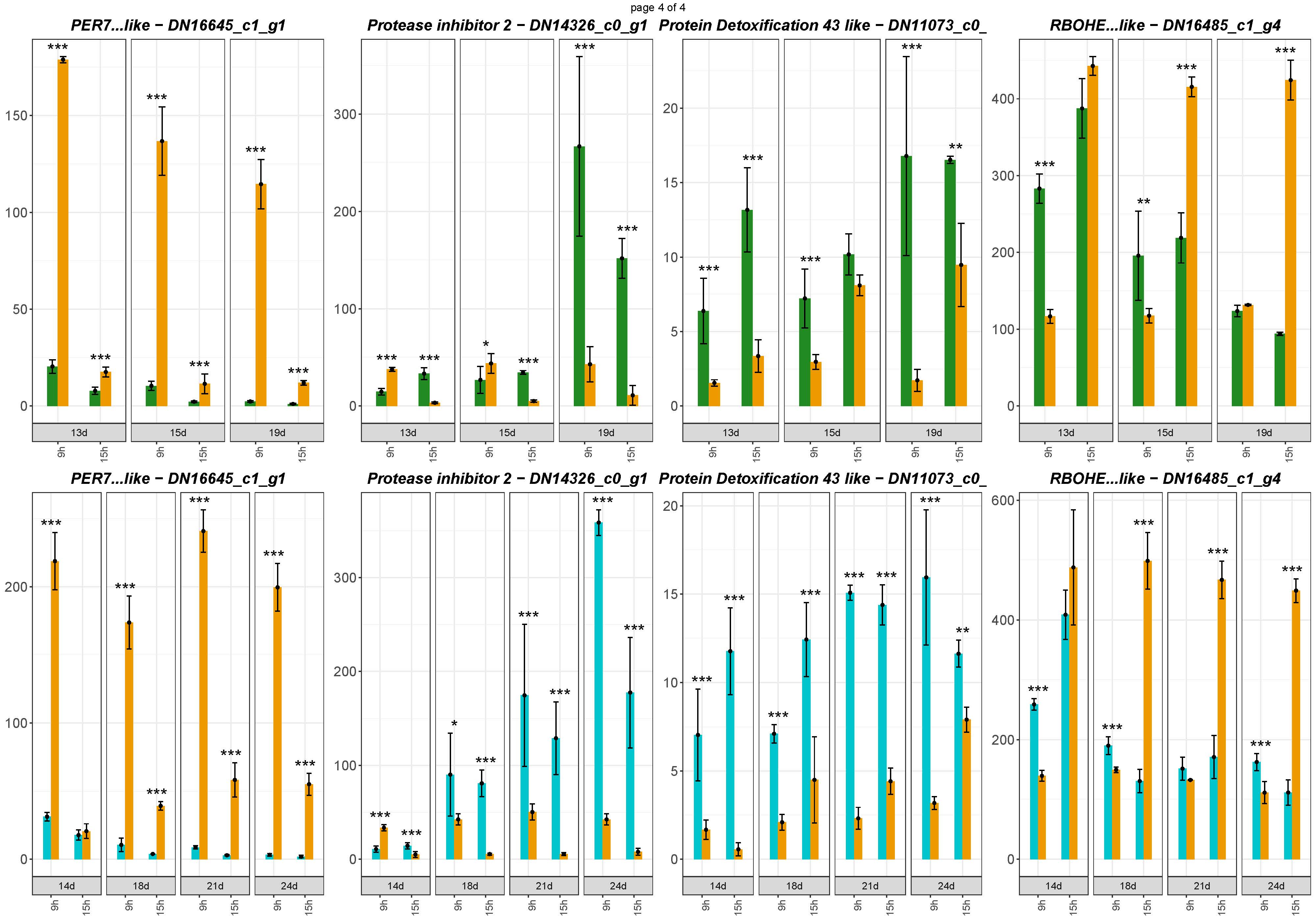

Supplement: Suppl_Figures_Storchova_jpeg.zip [file KPSB_A_2486083_SM1813.zip › Suppl_Figure6_Page_4.jpg]

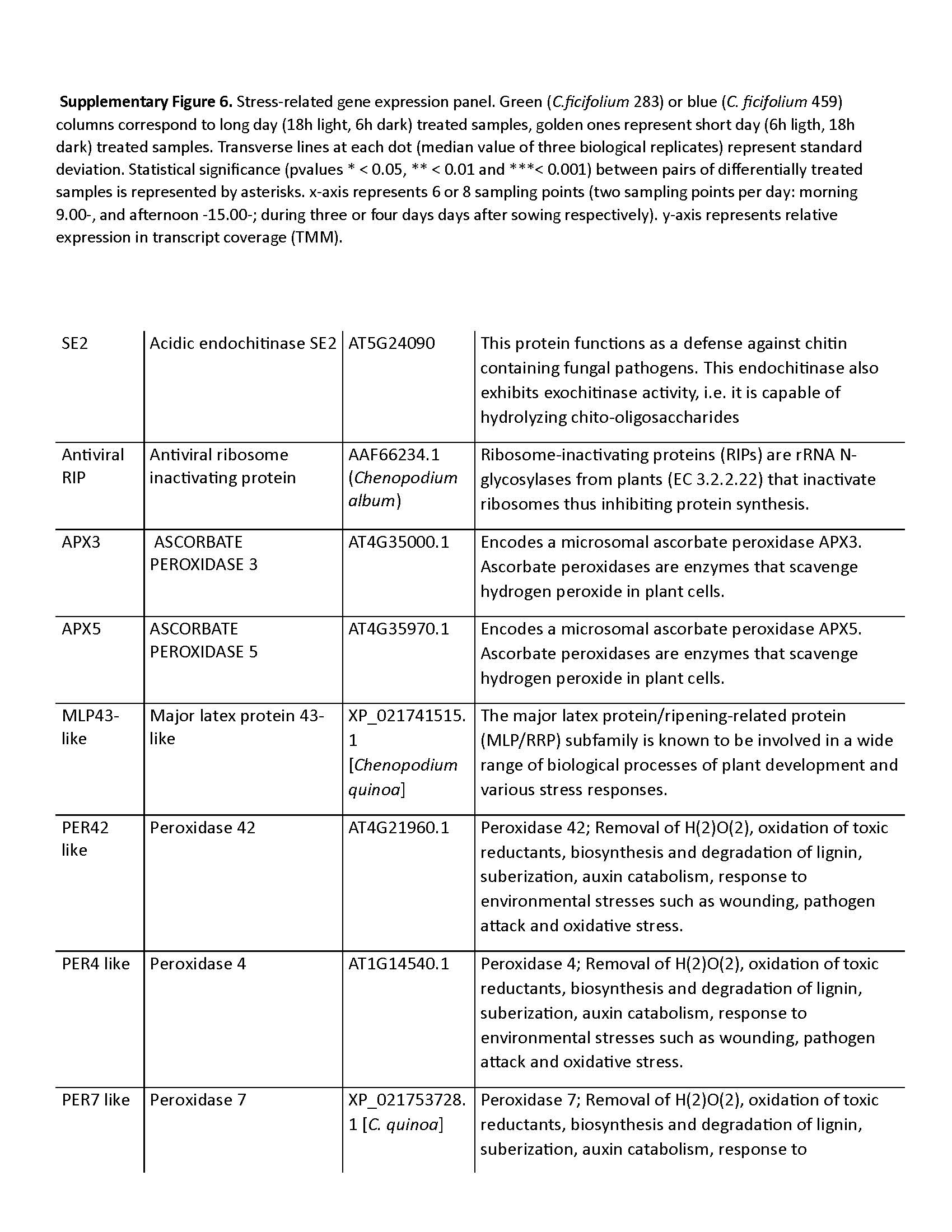

Supplement: Suppl_Figures_Storchova_jpeg.zip [file KPSB_A_2486083_SM1813.zip › Suppl_Figure6_Page_5.jpg]

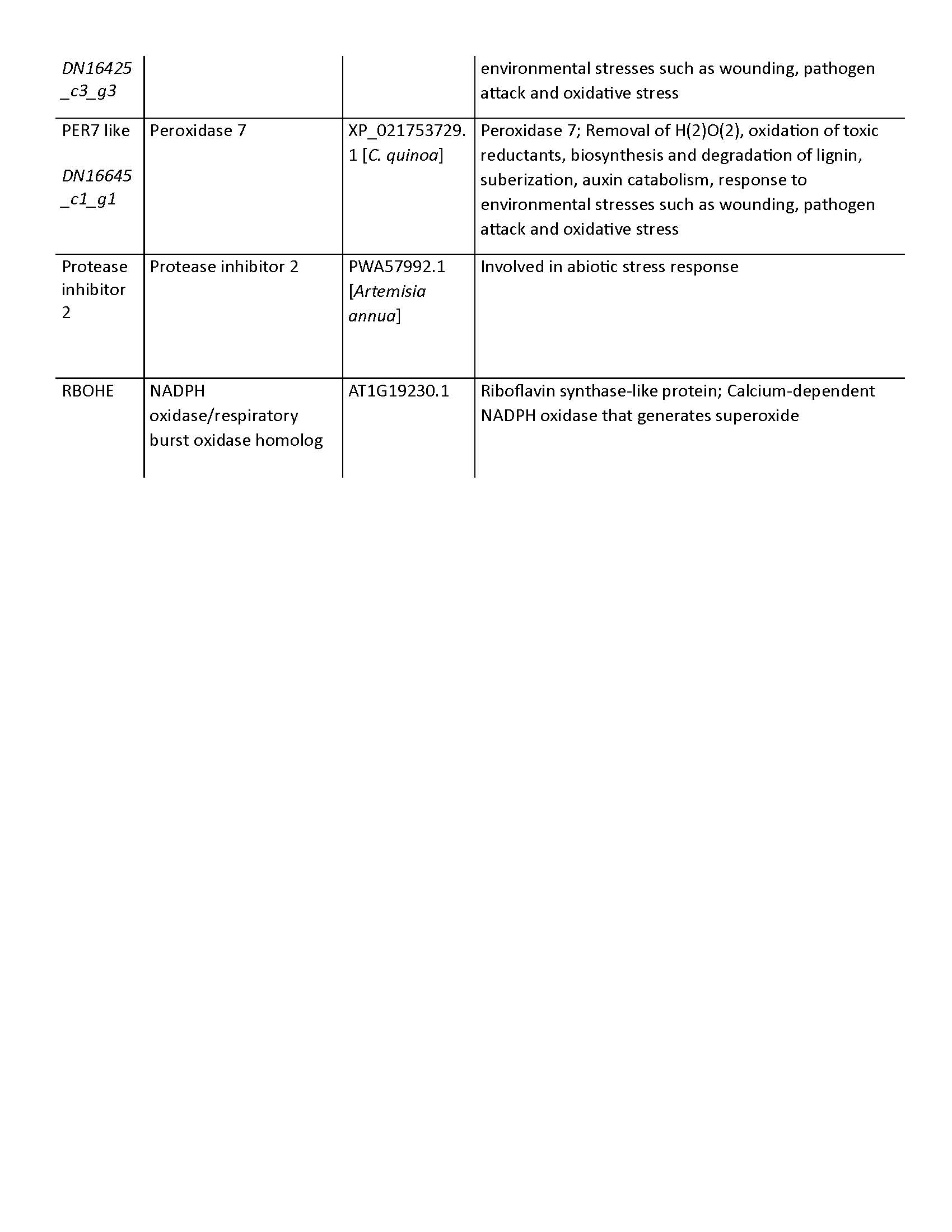

Supplement: Suppl_Figures_Storchova_jpeg.zip [file KPSB_A_2486083_SM1813.zip › Suppl_Figure6_Page_6.jpg]

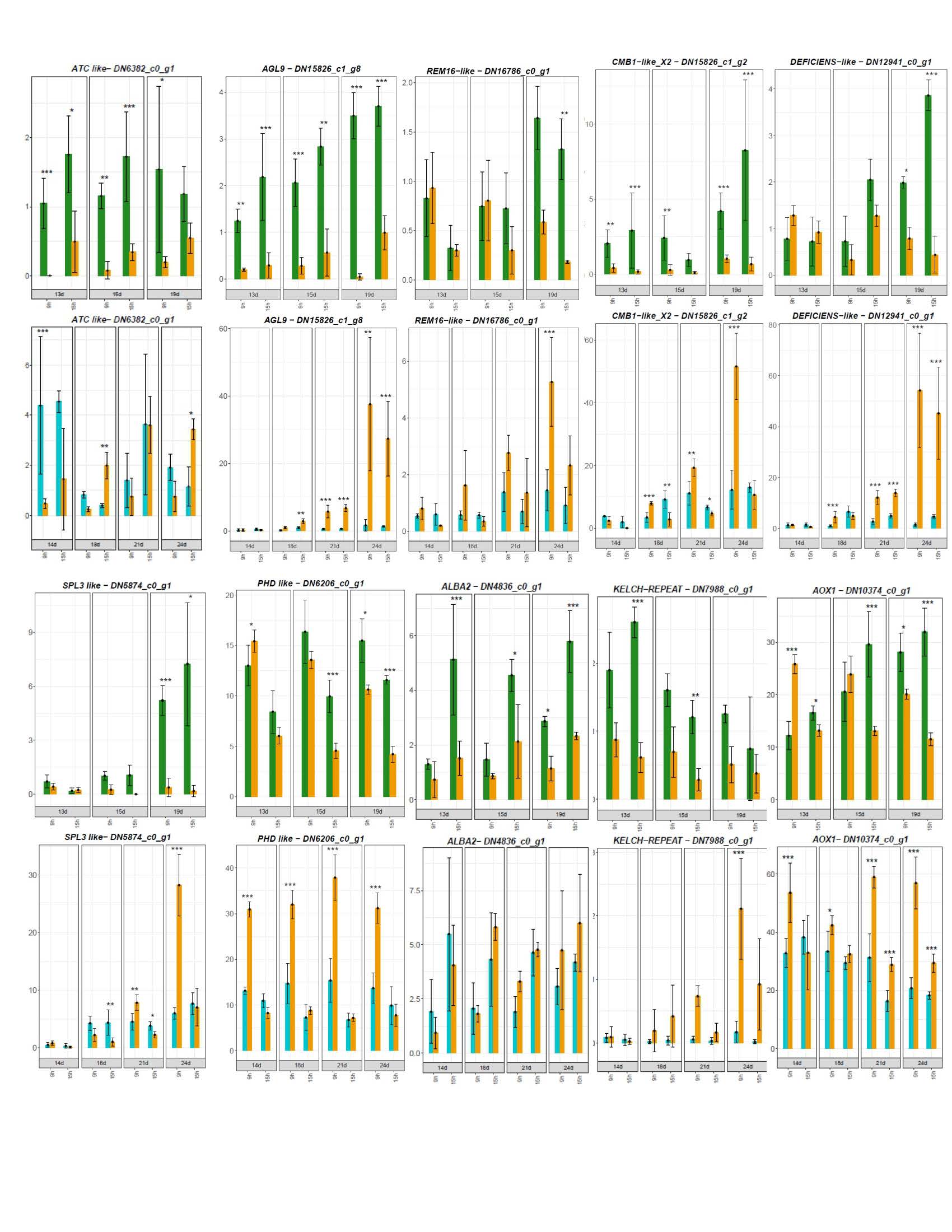

Supplement: Suppl_Figures_Storchova_jpeg.zip [file KPSB_A_2486083_SM1813.zip › Suppl_figure7_Page_1.jpg]

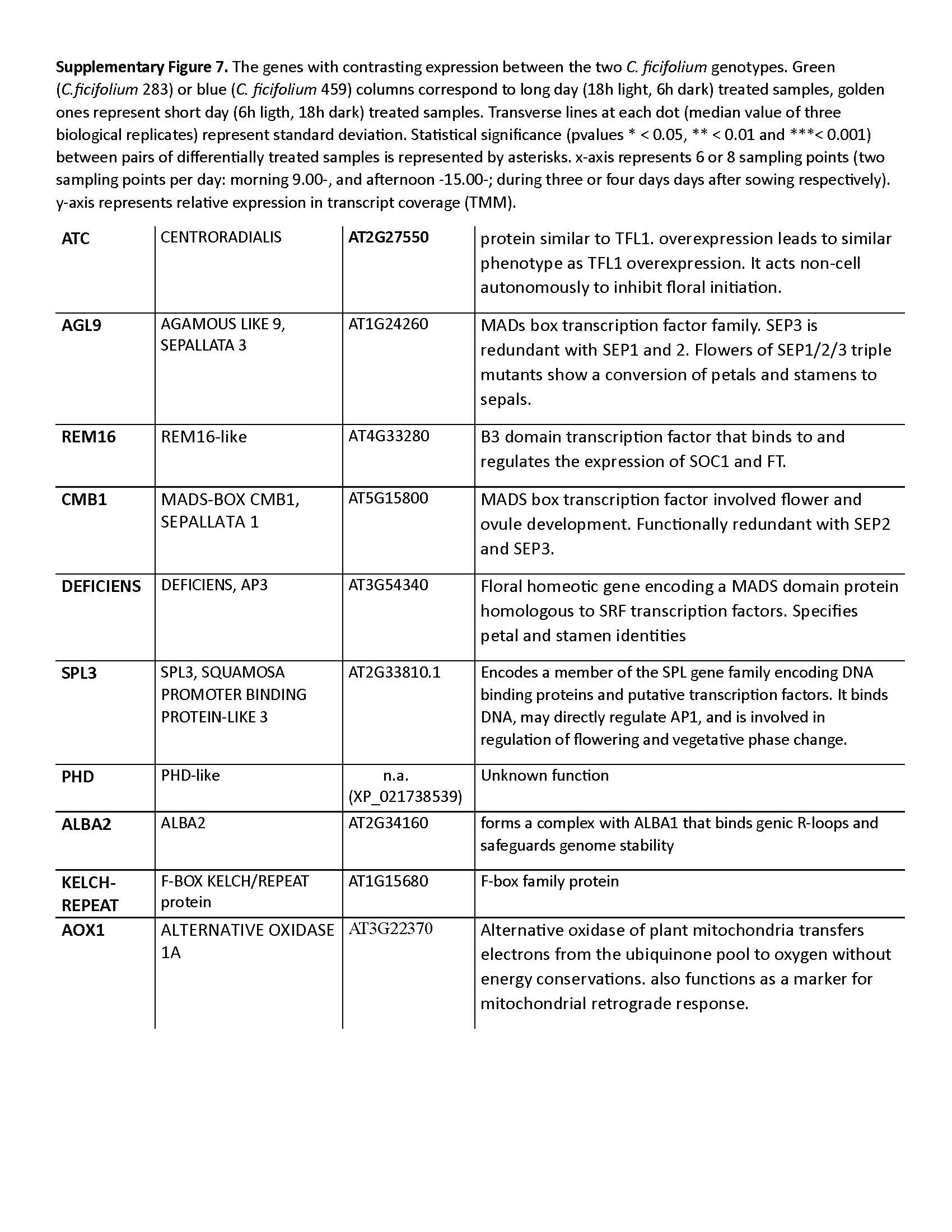

Supplement: Suppl_Figures_Storchova_jpeg.zip [file KPSB_A_2486083_SM1813.zip › Suppl_figure7_Page_2.jpg]

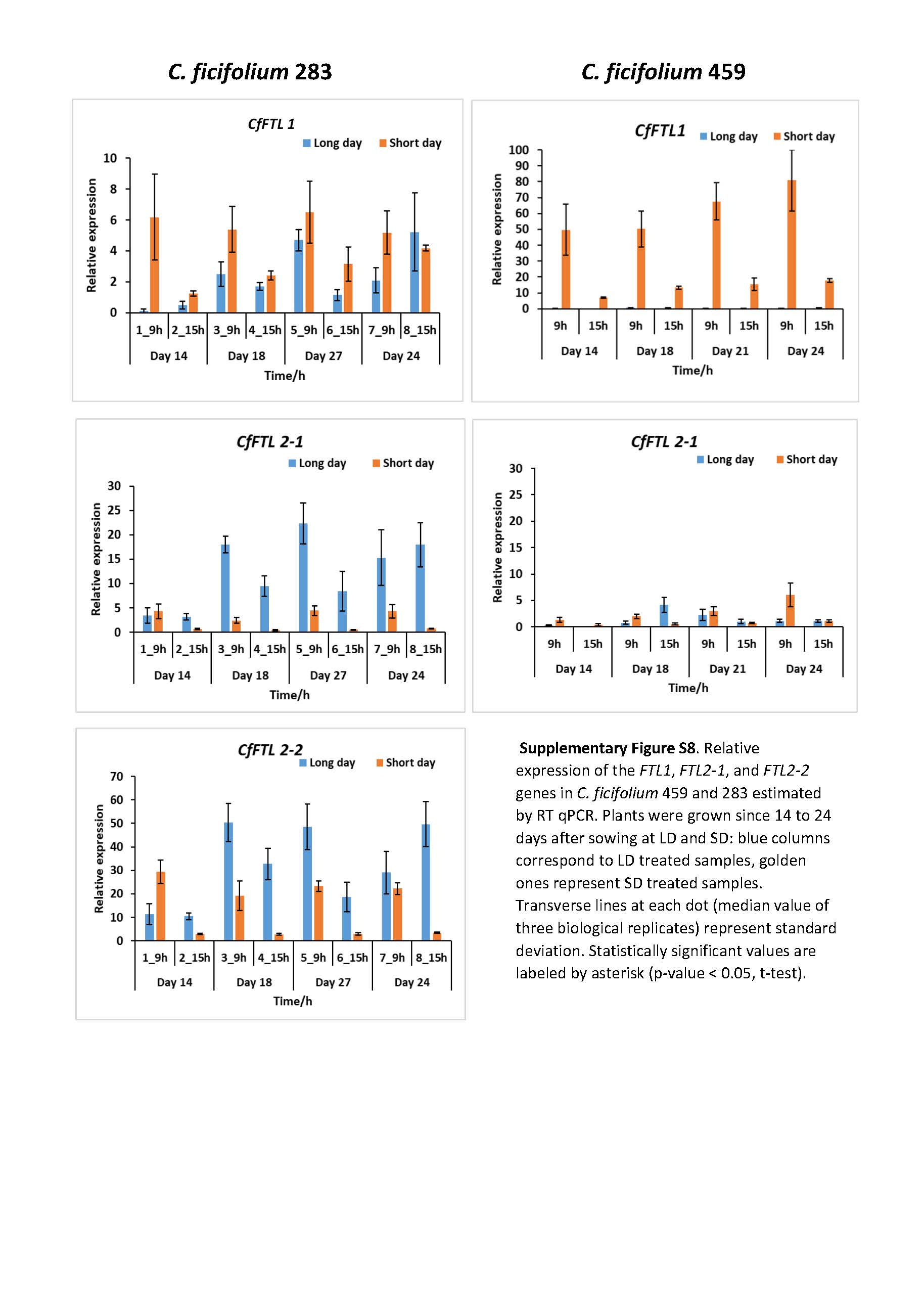

Supplement: Suppl_Figures_Storchova_jpeg.zip [file KPSB_A_2486083_SM1813.zip › Suppl_Figure8_qPCR graphs.jpg]

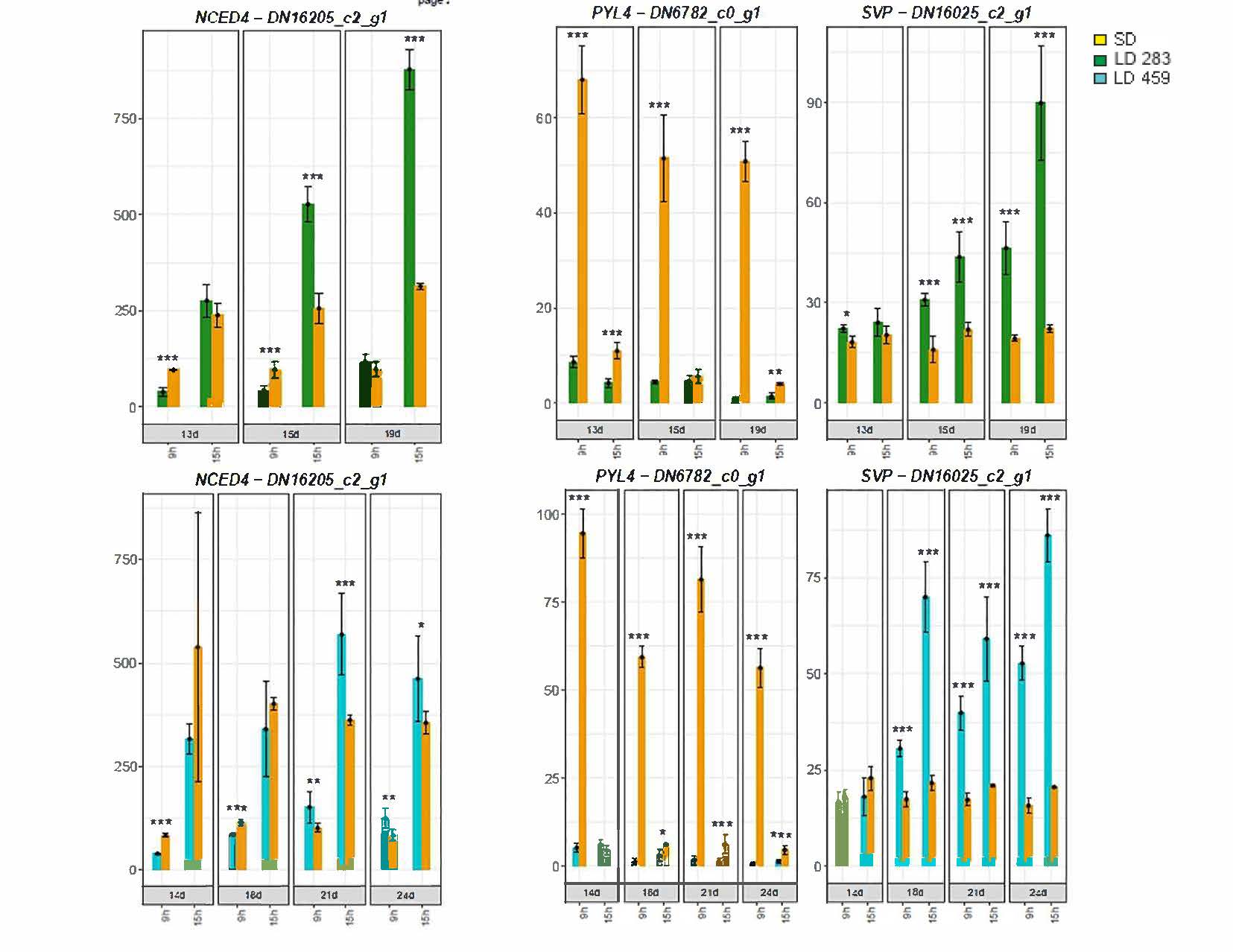

Supplement: Suppl_Figures_Storchova_jpeg.zip [file KPSB_A_2486083_SM1813.zip › Suppl_Figure1_Page_1.jpg]

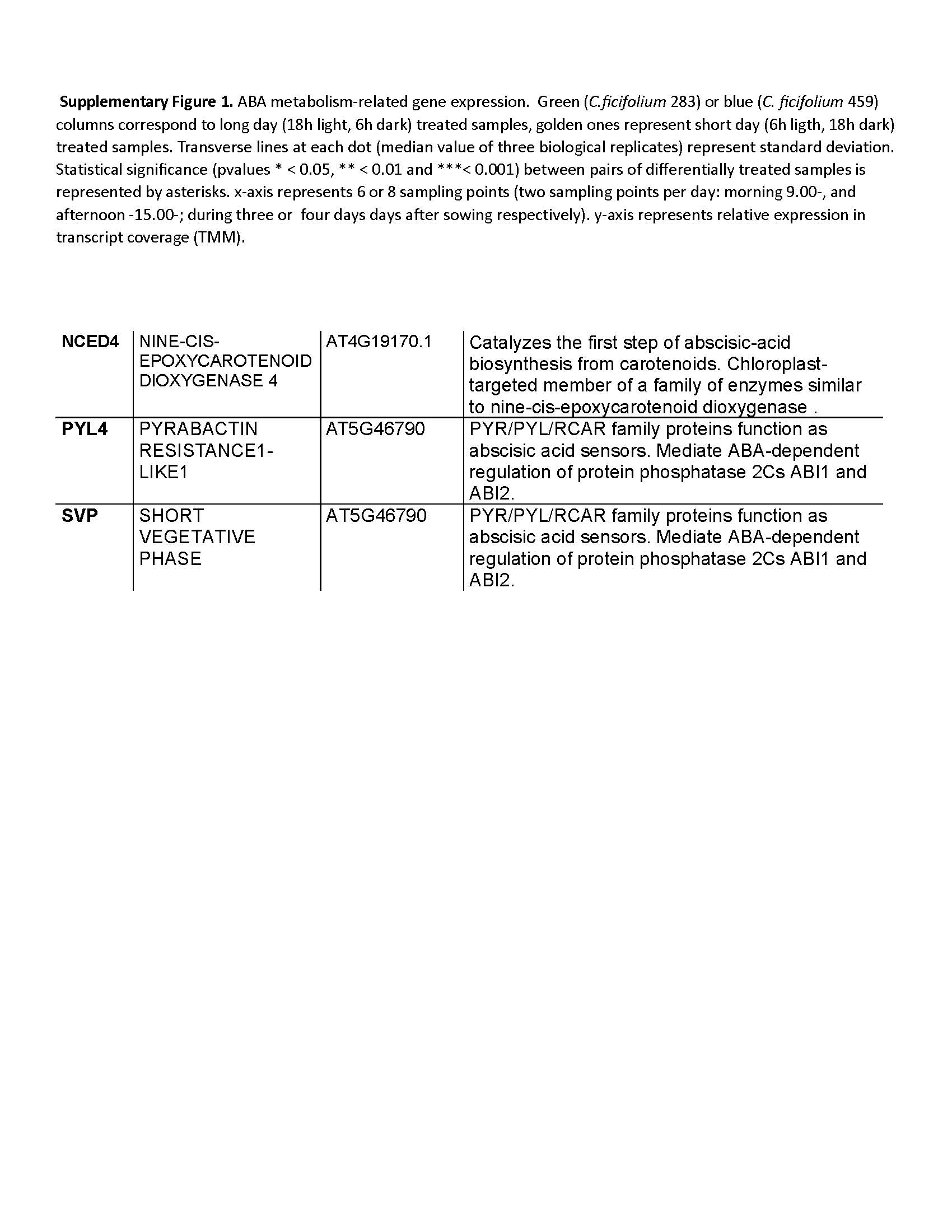

Supplement: Suppl_Figures_Storchova_jpeg.zip [file KPSB_A_2486083_SM1813.zip › Suppl_Figure1_Page_2.jpg]

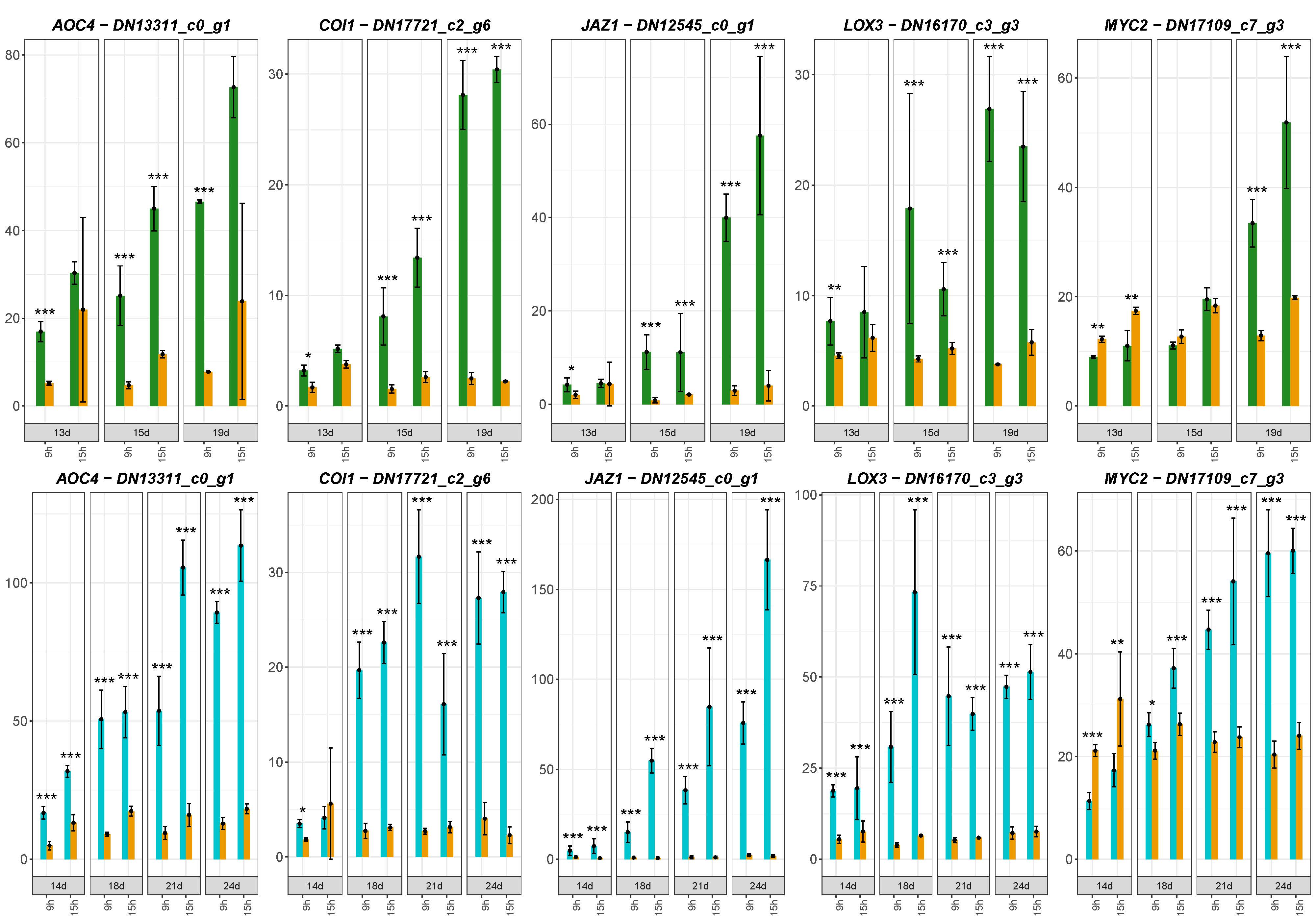

Supplement: Suppl_Figures_Storchova_jpeg.zip [file KPSB_A_2486083_SM1813.zip › Suppl_figure2_Page_1.jpg]

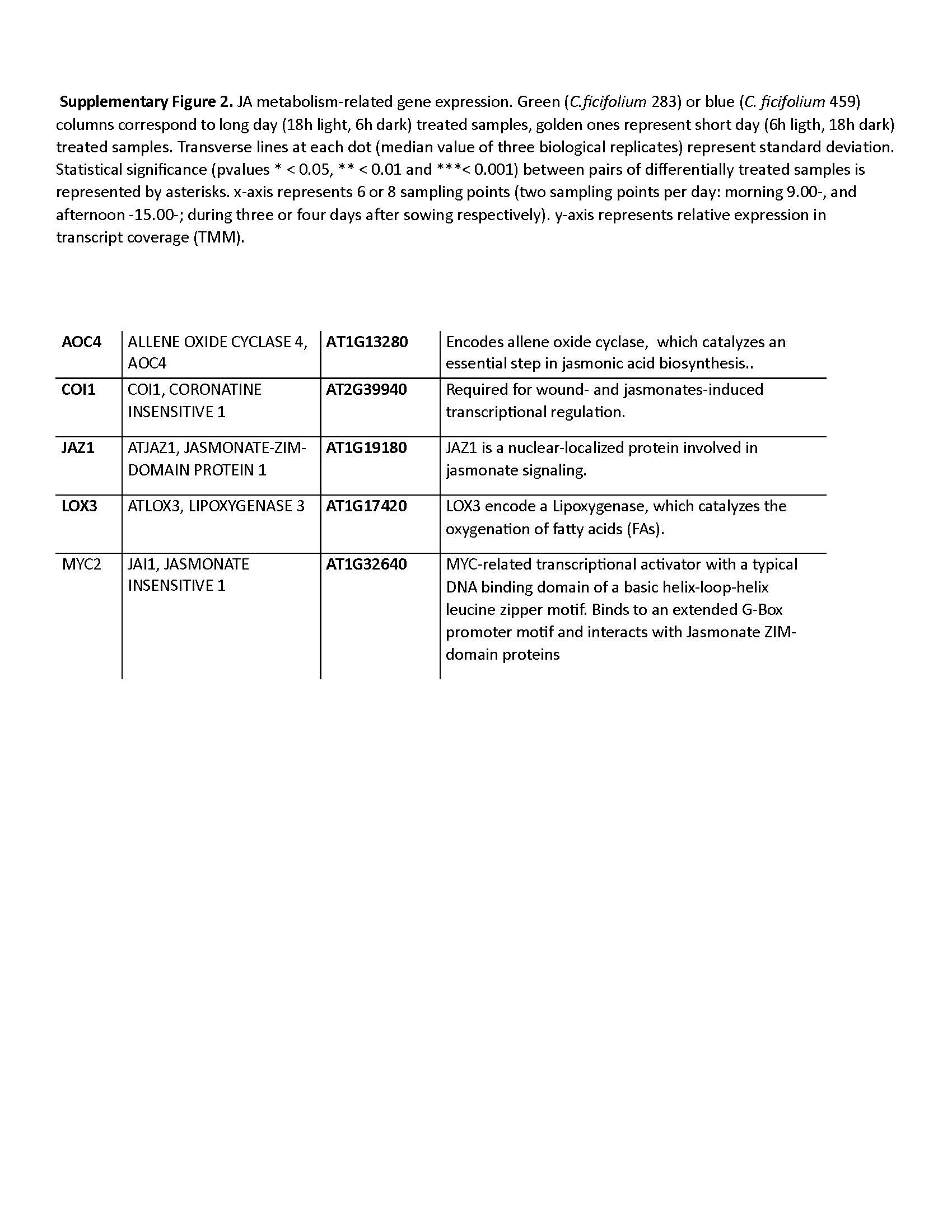

Supplement: Suppl_Figures_Storchova_jpeg.zip [file KPSB_A_2486083_SM1813.zip › Suppl_figure2_Page_2.jpg]

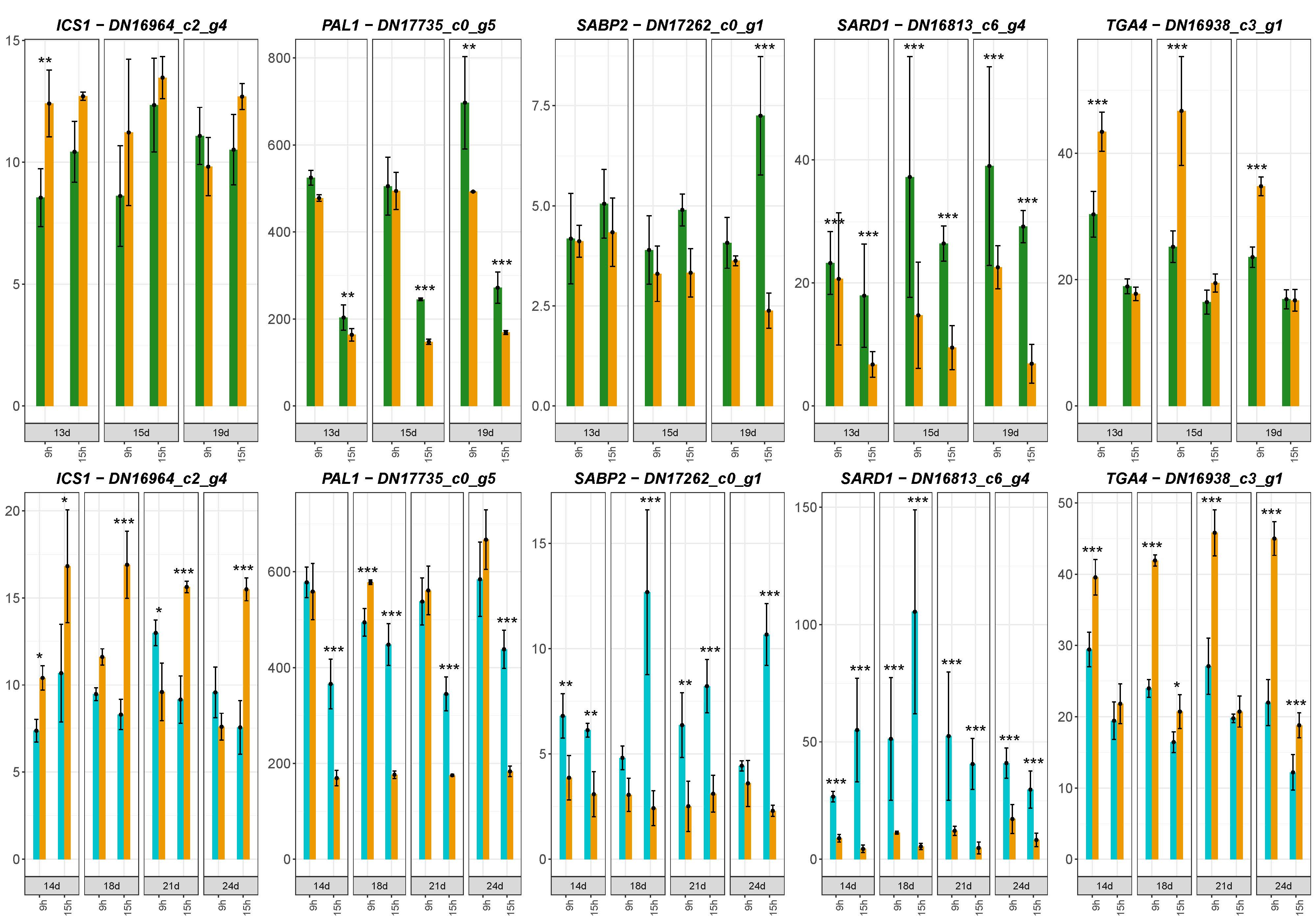

Supplement: Suppl_Figures_Storchova_jpeg.zip [file KPSB_A_2486083_SM1813.zip › Suppl_figure3_Page_1.jpg]

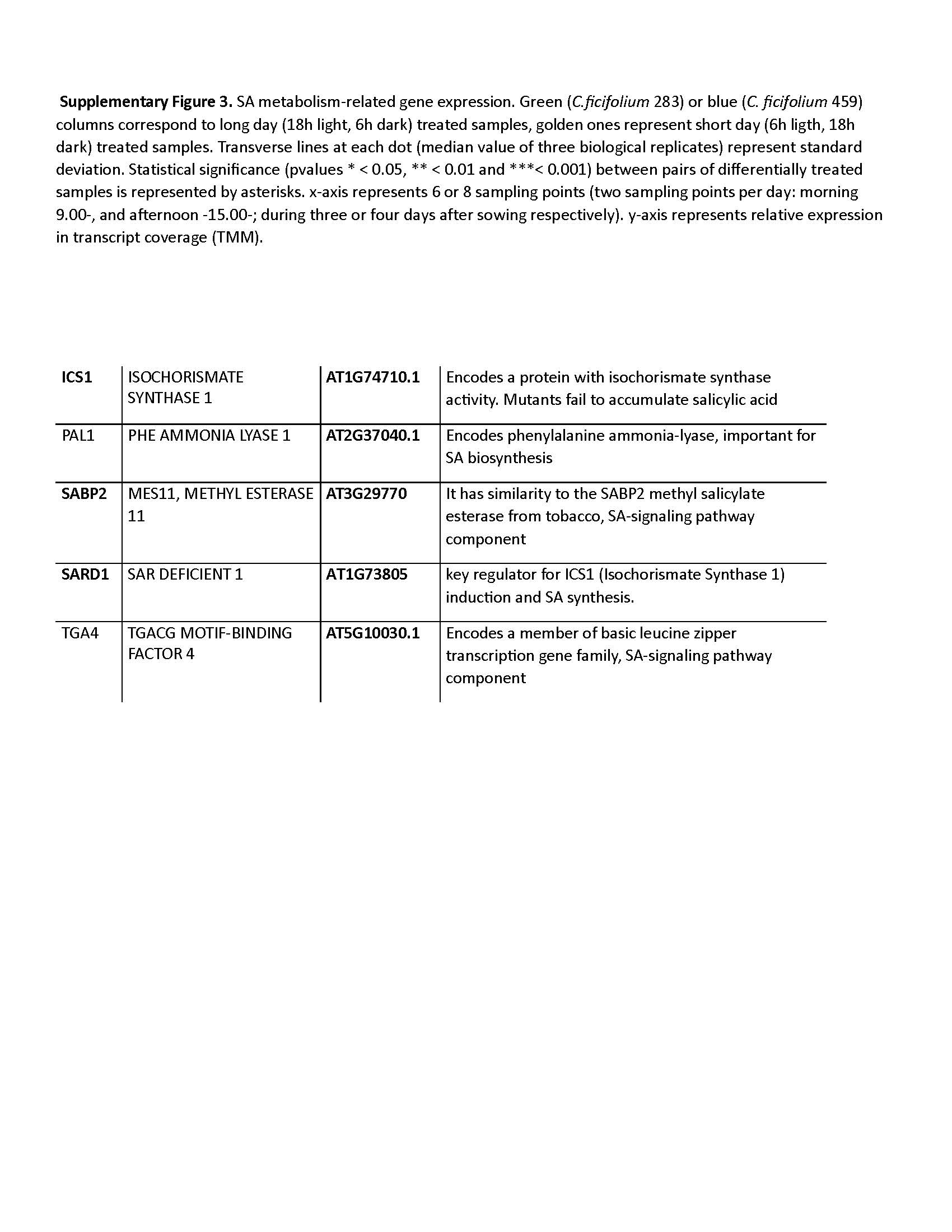

Supplement: Suppl_Figures_Storchova_jpeg.zip [file KPSB_A_2486083_SM1813.zip › Suppl_figure3_Page_2.jpg]

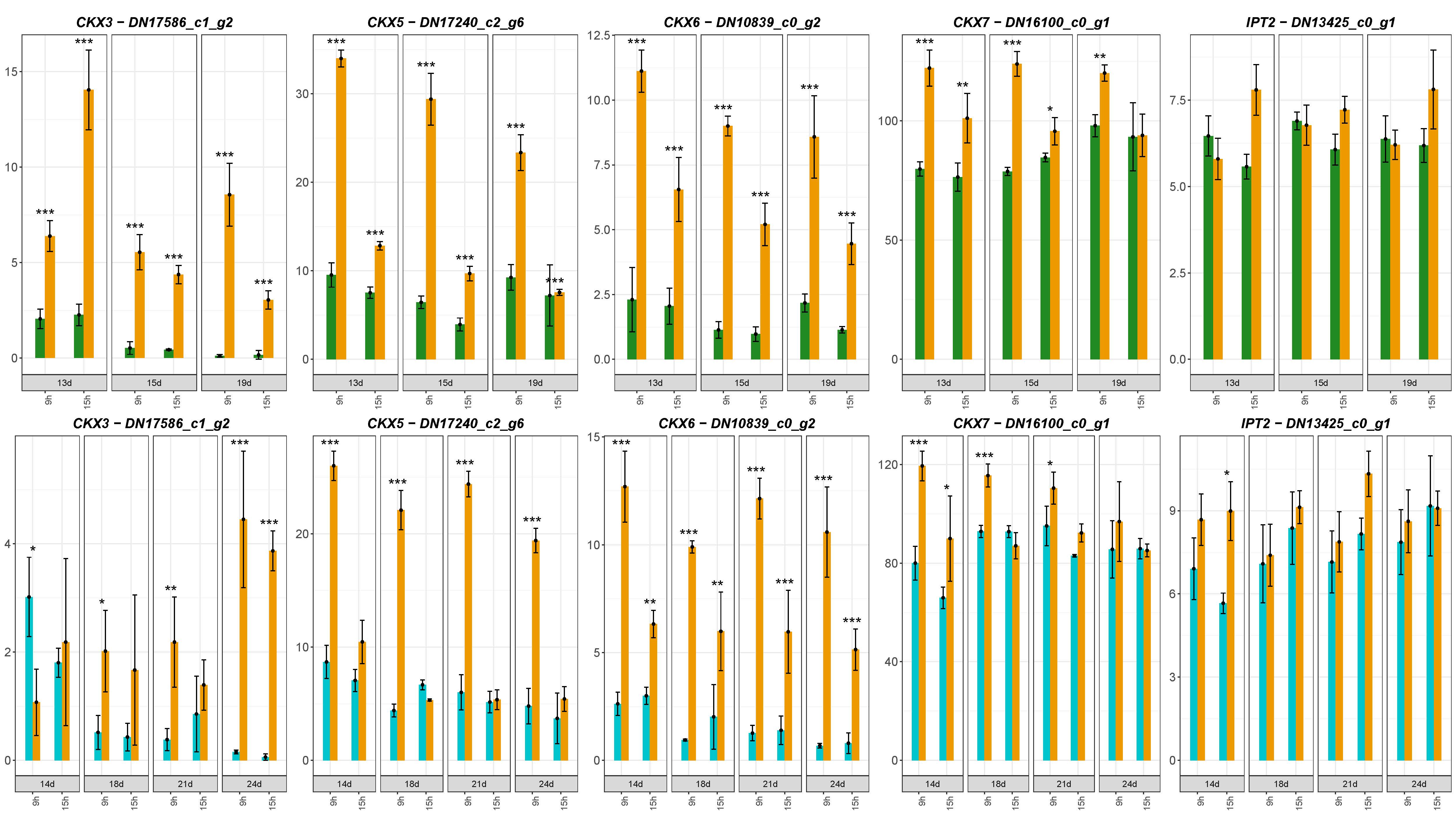

Supplement: Suppl_Figures_Storchova_jpeg.zip [file KPSB_A_2486083_SM1813.zip › Suppl_Figure4_Page_1.jpg]

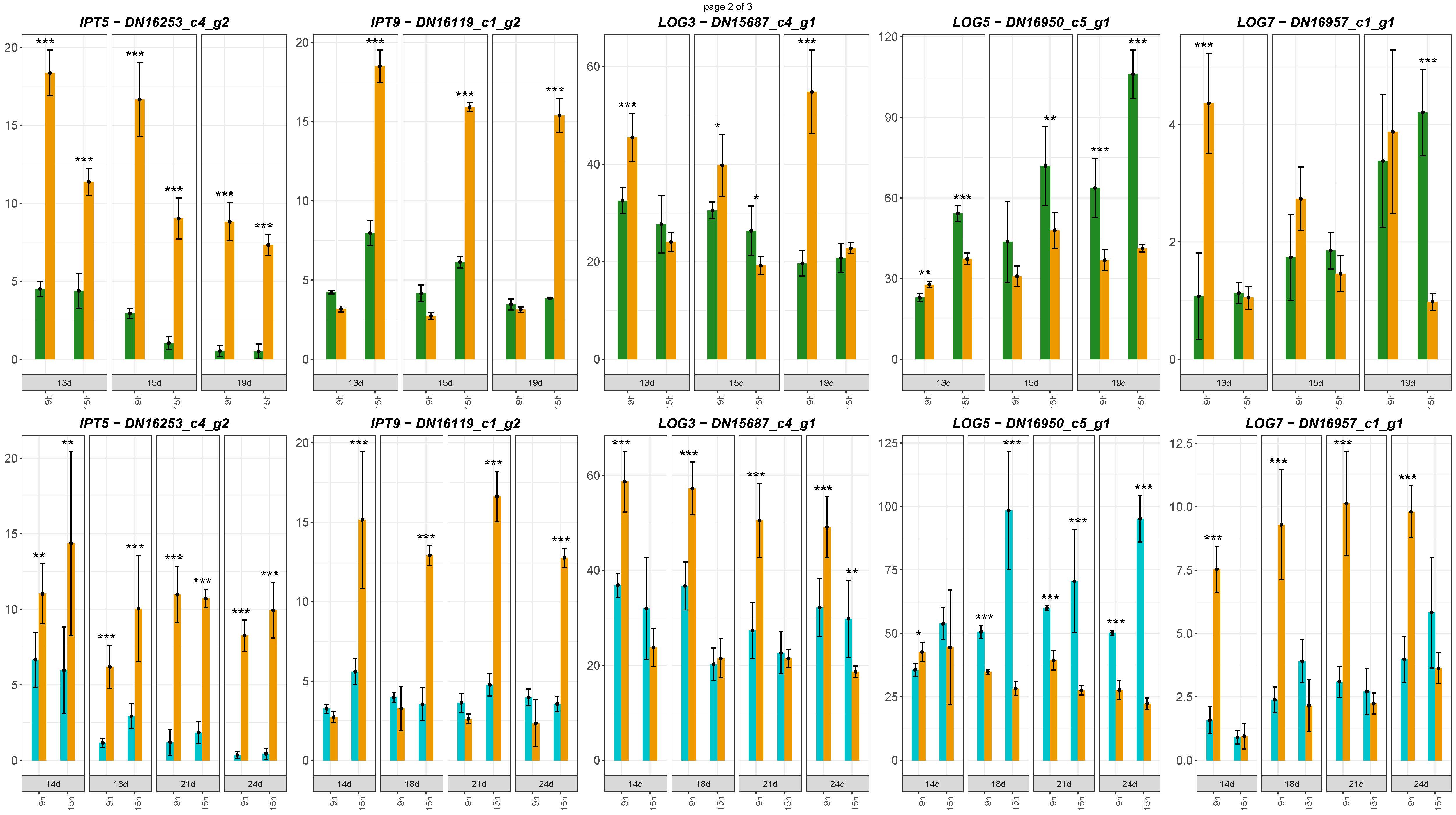

Supplement: Suppl_Figures_Storchova_jpeg.zip [file KPSB_A_2486083_SM1813.zip › Suppl_Figure4_Page_2.jpg]

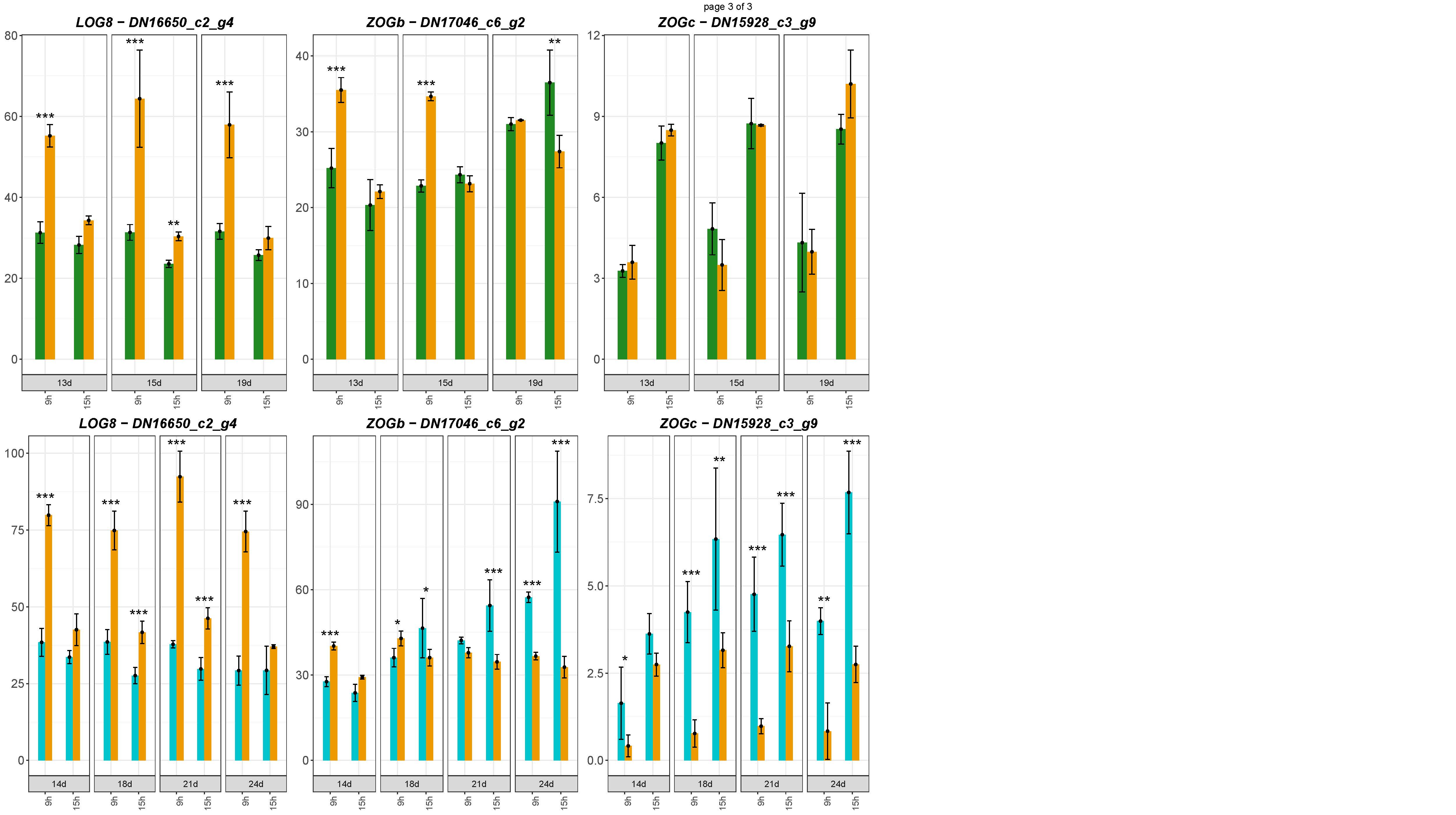

Supplement: Suppl_Figures_Storchova_jpeg.zip [file KPSB_A_2486083_SM1813.zip › Suppl_Figure4_Page_3.jpg]

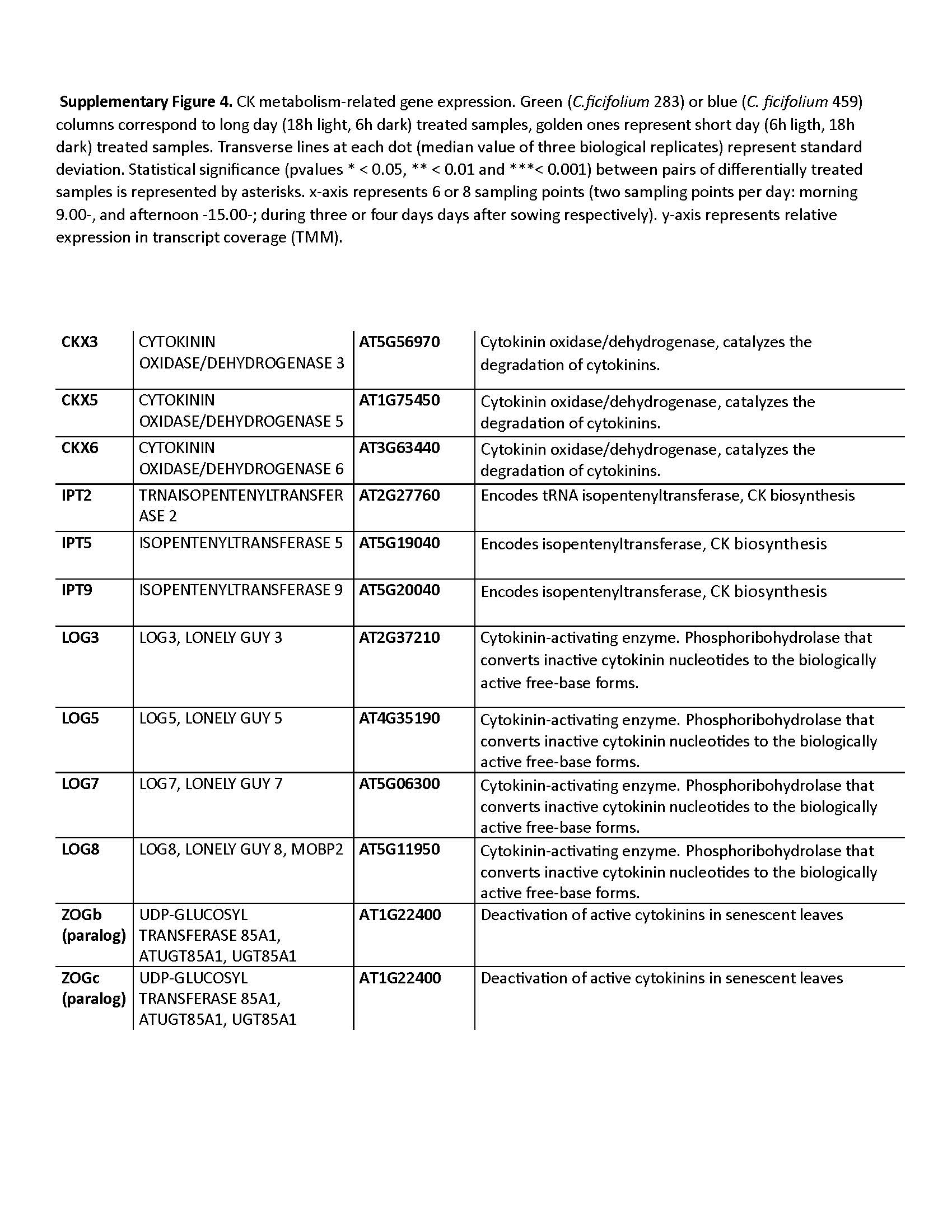

Supplement: Suppl_Figures_Storchova_jpeg.zip [file KPSB_A_2486083_SM1813.zip › Suppl_Figure4_Page_4.jpg]

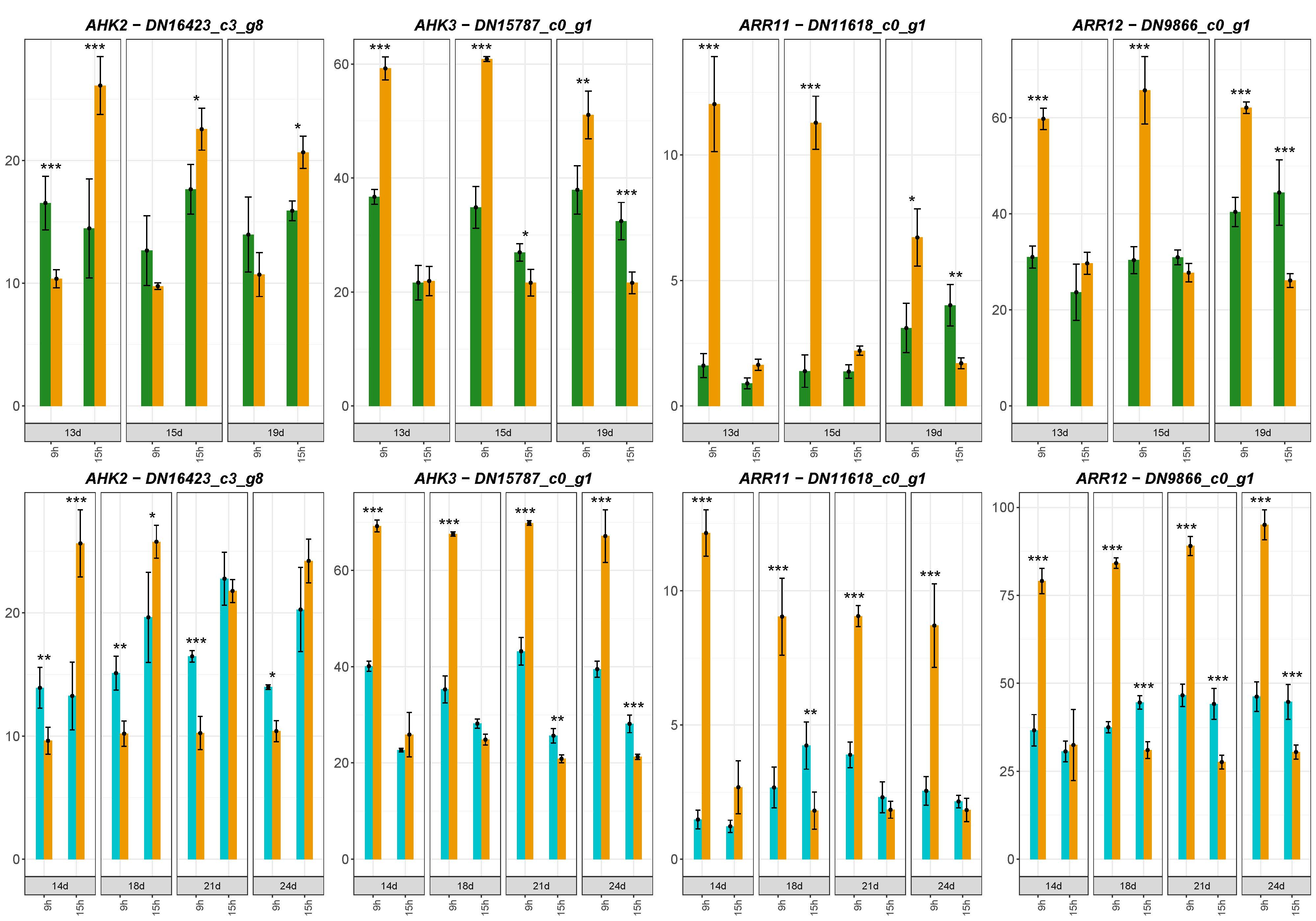

Supplement: Suppl_Figures_Storchova_jpeg.zip [file KPSB_A_2486083_SM1813.zip › Suppl_Figure5_Page_1.jpg]

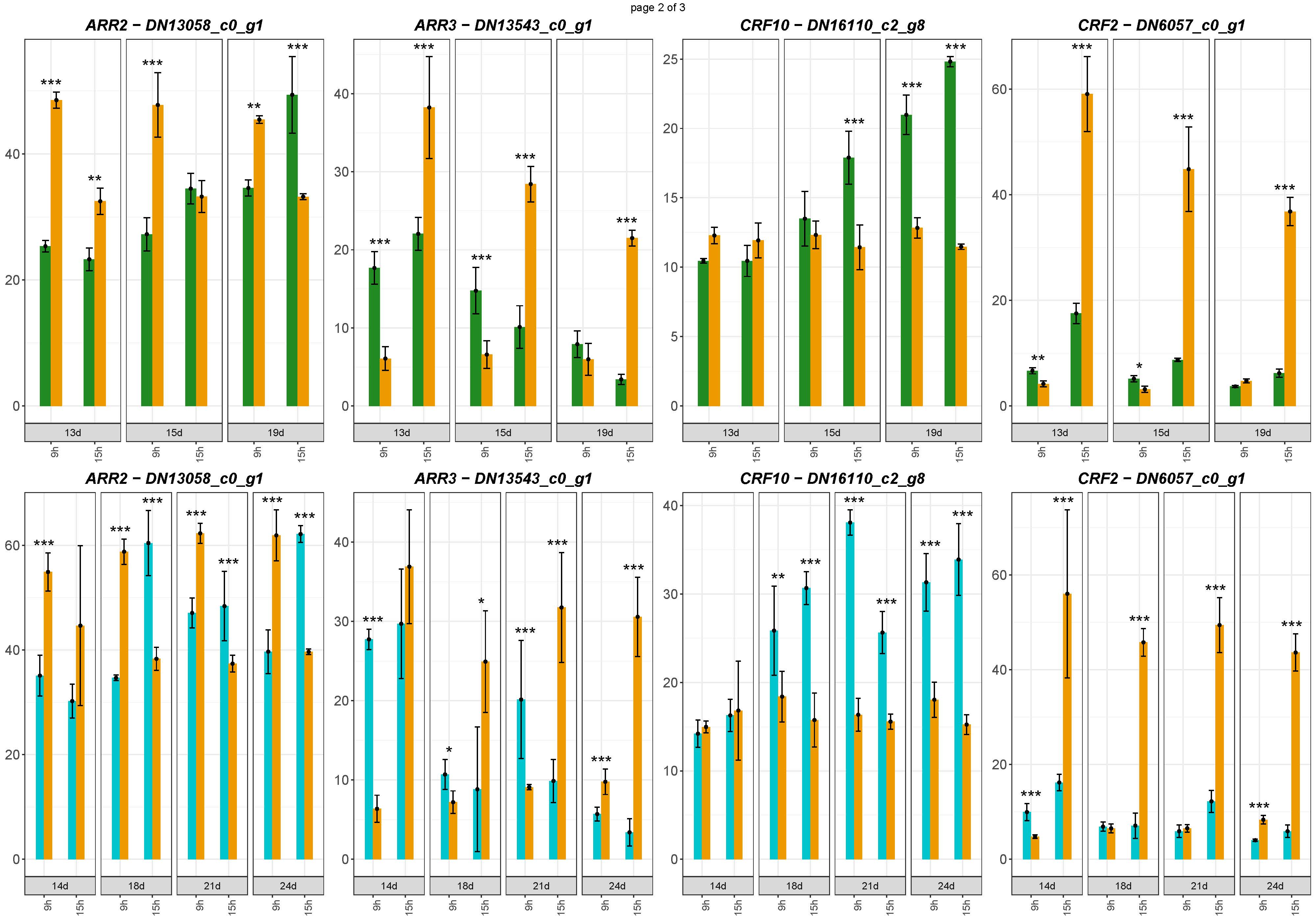

Supplement: Suppl_Figures_Storchova_jpeg.zip [file KPSB_A_2486083_SM1813.zip › Suppl_Figure5_Page_2.jpg]

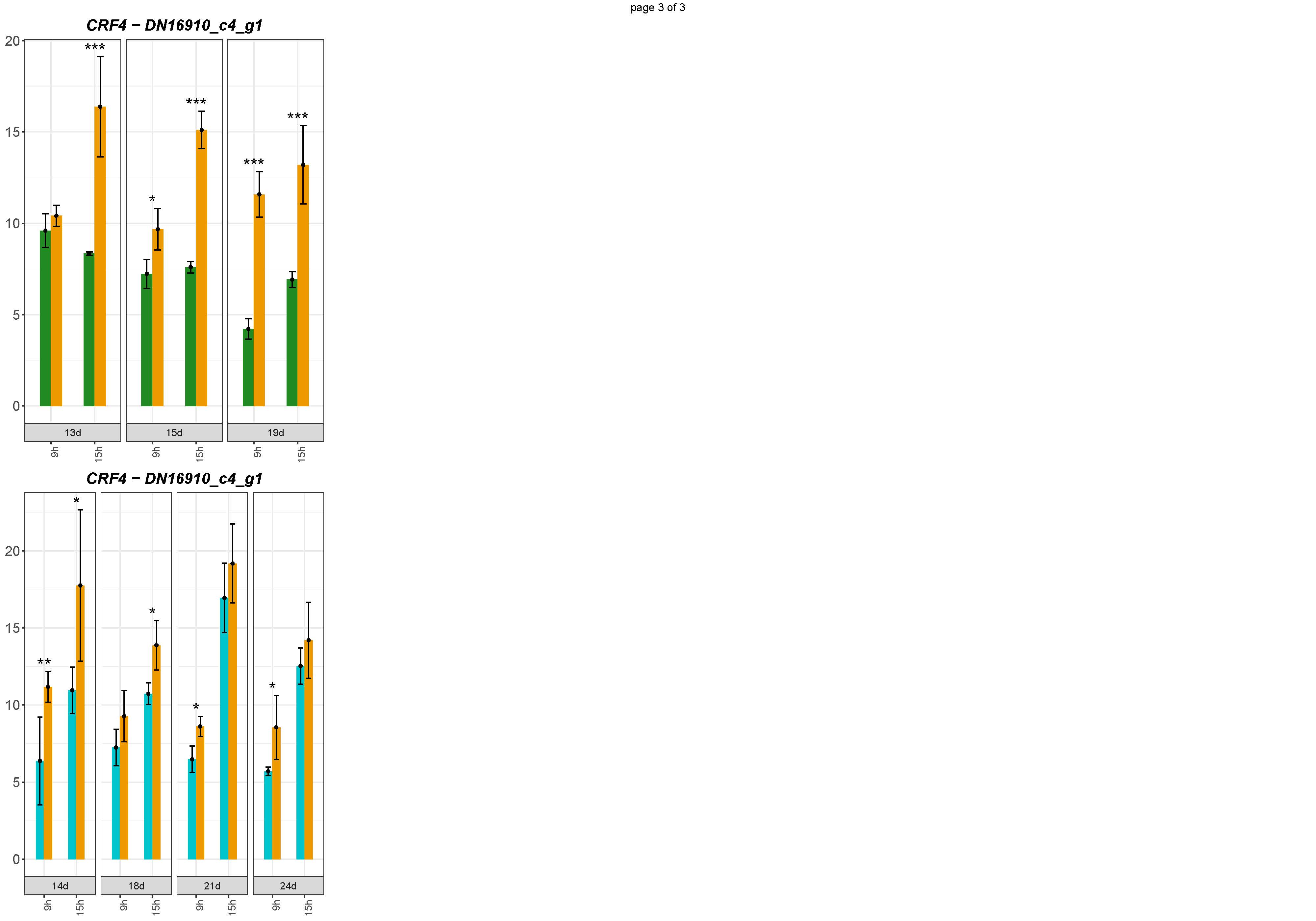

Supplement: Suppl_Figures_Storchova_jpeg.zip [file KPSB_A_2486083_SM1813.zip › Suppl_Figure5_Page_3.jpg]

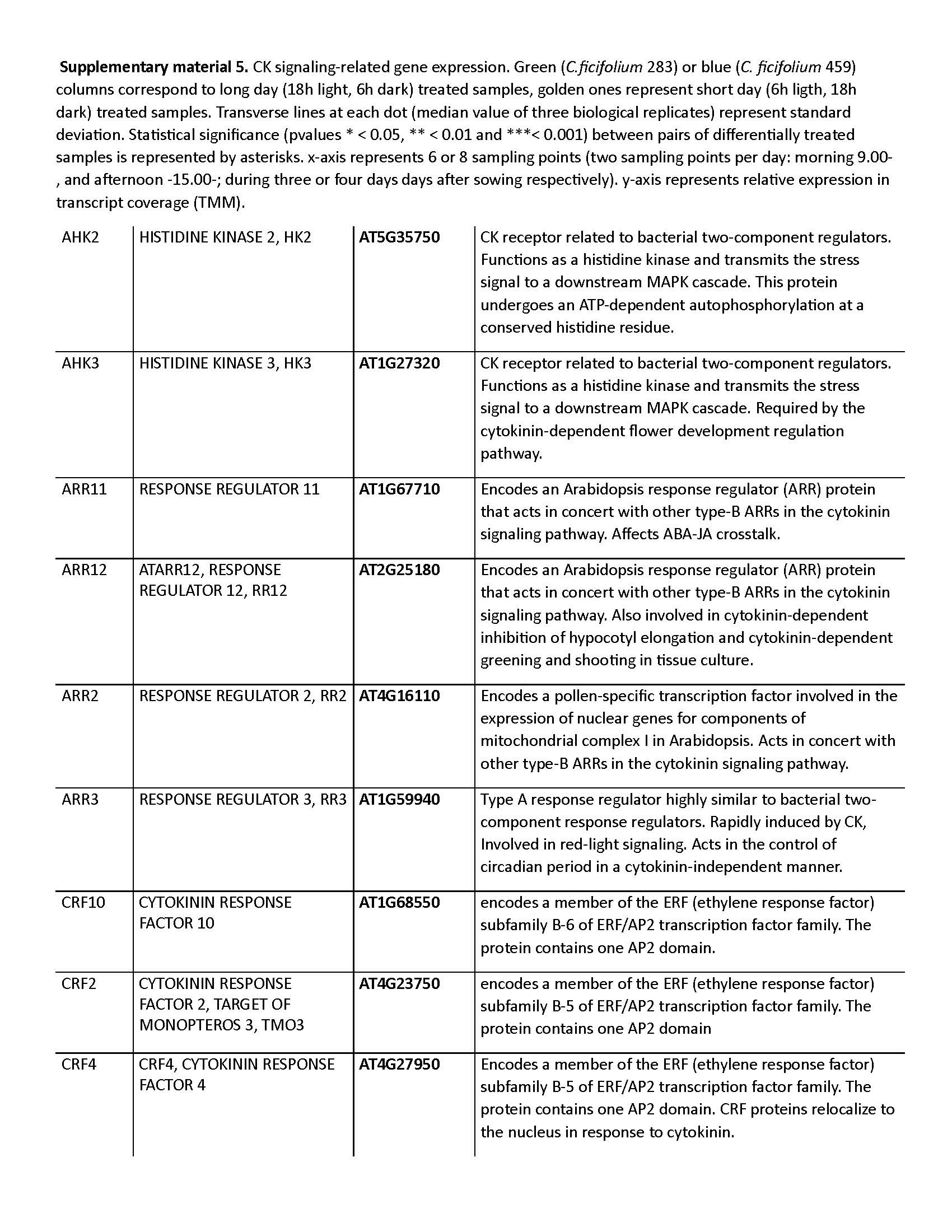

Supplement: Suppl_Figures_Storchova_jpeg.zip [file KPSB_A_2486083_SM1813.zip › Suppl_Figure5_Page_4.jpg]

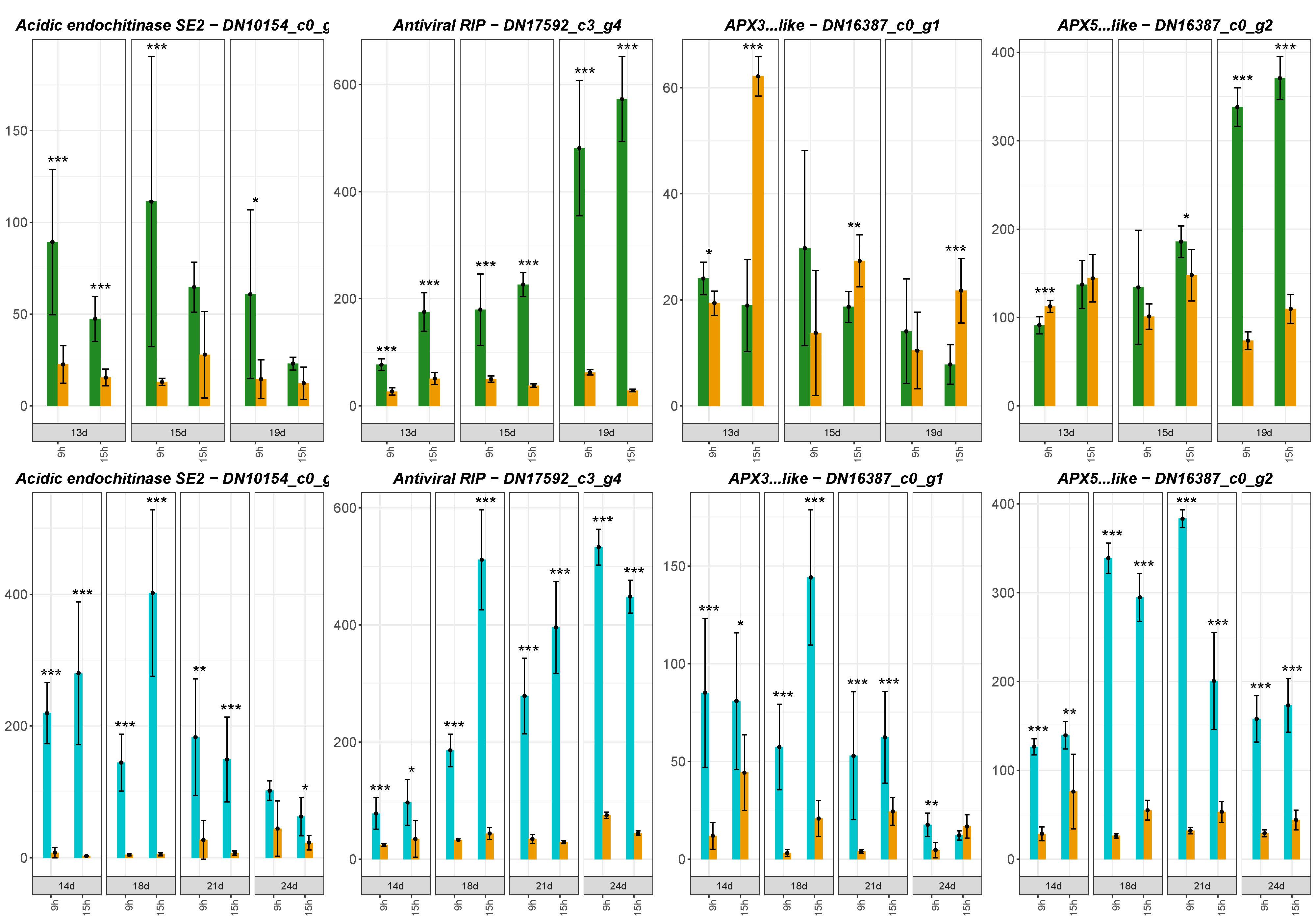

Supplement: Suppl_Figures_Storchova_jpeg.zip [file KPSB_A_2486083_SM1813.zip › Suppl_Figure6_Page_1.jpg]
